# Associations between Single Nucleotide Polymorphisms in Iron-Related Genes and Iron Status in Multiethnic Populations

**DOI:** 10.1371/journal.pone.0038339

**Published:** 2012-06-22

**Authors:** Christine E. McLaren, Stela McLachlan, Chad P. Garner, Chris D. Vulpe, Victor R. Gordeuk, John H. Eckfeldt, Paul C. Adams, Ronald T. Acton, Joseph A. Murray, Catherine Leiendecker-Foster, Beverly M. Snively, Lisa F. Barcellos, James D. Cook, Gordon D. McLaren

**Affiliations:** 1 Department of Epidemiology, University of California Irvine, Irvine, California, United States of America; 2 Nutritional Sciences and Toxicology, University of California, Berkeley, Berkeley, California, United States of America; 3 Department of Medicine, Sickle Cell Center, University of Illinois at Chicago, Chicago, Illinois, United States of America; 4 Department of Laboratory Medicine and Pathology, University of Minnesota, Minneapolis, Minnesota, United States of America; 5 London Health Sciences Centre, London, Ontario, Canada; 6 Department of Microbiology, University of Alabama at Birmingham, Birmingham, Alabama, United States of America; 7 Division of Gastroenterology/Hepatology, Mayo Clinic College of Medicine, Rochester, Minnesota, United States of America; 8 Department of Biostatistical Sciences, Division of Public Health Sciences, Wake Forest University School of Medicine, Winston-Salem, North Carolina, United States of America; 9 School of Public Health, University of California, Berkeley, Berkeley, California, United States of America; 10 Department of Medicine, The University of Kansas Medical Center, Kansas City, Kansas, United States of America; 11 Department of Veterans Affairs Long Beach Healthcare System, Long Beach, California, United States of America; 12 Division of Hematology/Oncology, Department of Medicine, University of California Irvine, Irvine, California, United States of America; Institut national de la santé et de la recherche médicale (INSERM), France

## Abstract

The existence of multiple inherited disorders of iron metabolism suggests genetic contributions to iron deficiency. We previously performed a genome-wide association study of iron-related single nucleotide polymorphisms (SNPs) using DNA from white men aged ≥25 y and women ≥50 y in the Hemochromatosis and Iron Overload Screening (HEIRS) Study with serum ferritin (SF) ≤12 µg/L (cases) and controls (SF >100 µg/L in men, SF >50 µg/L in women). We report a follow-up study of white, African-American, Hispanic, and Asian HEIRS participants, analyzed for association between SNPs and eight iron-related outcomes. Three chromosomal regions showed association across multiple populations, including SNPs in the *TF* and *TMPRSS6* genes, and on chromosome 18q21. A novel SNP rs1421312 in *TMPRSS6* was associated with serum iron in whites (p = 3.7×10^−6^) and replicated in African Americans (p = 0.0012).Twenty SNPs in the *TF* gene region were associated with total iron-binding capacity in whites (p<4.4×10^−5^); six SNPs replicated in other ethnicities (p<0.01). SNP rs10904850 in the *CUBN* gene on 10p13 was associated with serum iron in African Americans (P = 1.0×10^−5^). These results confirm known associations with iron measures and give unique evidence of their role in different ethnicities, suggesting origins in a common founder.

## Introduction

The levels of iron, a micronutrient required for life and health, must be tightly regulated to avoid excess unbound iron that can generate toxic free radicals while at the same time maintaining adequate supplies for vital functions including oxygen carrying capacity [1], [2]. Disorders of iron metabolism underlie some of the most prevalent diseases in humans and encompass a broad spectrum of clinical manifestations, ranging from anemia to iron overload and neurodegenerative diseases [3]. Body iron balance normally is maintained by control of iron uptake from the diet by the duodenal enterocytes and its transfer to the systemic circulation, as humans cannot actively excrete iron. Intestinal iron absorption and release of stored iron from macrophages are dependent on similar pathways [4]. While many factors can lead to iron deficiency, most commonly it is attributable to blood loss, a lack of dietary abundance, or defective absorption that collectively affect two-thirds of the world’s population [5]. However, the existence of multiple inherited disorders of iron metabolism in man, rodents and other vertebrates make plausible a genetic contribution to iron deficiency [6], [7], [8]. It is widely known that genetics can play a significant role in the iron overload found in whites, the most common example being hereditary hemochromatosis attributable to mutations in the hemochromatosis gene, *HFE* [9], [10]. Less is known about the role of genetic factors in disorders of iron status in other ethnic groups or about genetic effects on susceptibility to iron deficiency.

In order to investigate the genetic contribution to iron deficiency in whites, we recently completed a genome-wide association study (GWAS) in iron-deficient white male and female participants in the Hemochromatosis and Iron Overload Screening (HEIRS) study [11]. Case-control status and seven quantitative iron-related measures were studied, including serum iron (SI), total iron-binding capacity (TIBC), unsaturated iron-binding capacity (UIBC), transferrin saturation (TfS), serum ferritin concentration (SF), serum transferrin receptor (sTfR), and body iron. The quantitative iron-related measures were significantly associated with presence of iron deficiency in the GWAS (p<0.001), and a high degree of concordance was observed in the results across the quantitative traits. The study found genome-wide statistically significant associations between at least one of the iron measures and SNPs on chromosomes 2p14, 3q22 (in the transferrin gene, *TF*), 6p22 (in the *HFE* gene), 7p21 and 22q11, with the association at *TF* being replicated in a follow-up case-control study.

Furthermore, mutations in the *TMPRSS6* gene have been implicated in iron deficiency anemia refractory to oral iron therapy within white populations [12], [13], [14]. Further evidence of genetic influences on iron status was found in a recent GWAS of four serum markers of iron status (serum iron, transferrin, transferrin saturation and serum ferritin) [15]. Along with confirming previously reported associations of the *HFE* C282Y mutation, significant associations between iron status markers and the *TMPRSS6* gene and *TF* were reported. Tanaka and colleagues investigated genetic variants associated with iron concentrations in persons not affected by overt genetic disorders of iron metabolism and found SNPs in *TMPRSS6* were strongly associated with lower serum iron concentration and other hematological variables [16]. Most of the genetic studies of iron deficiency and iron status markers carried out to date have used samples from populations of white individuals.

In the current study, we have investigated SNPs and iron status in multiple ethnic groups, including not only whites but also African Americans, Hispanics, and Asians. Our aims were to investigate whether the same SNPs associated with iron deficiency in whites play a similar role in other ethnic groups and to identify additional SNPs that may play a role in iron deficiency in these populations. The role of 1239 candidate or known SNPs associated with iron deficiency and iron status measures was tested in white, African-American, Hispanic and Asian iron deficient case and normal control samples. For consistency, the same outcome measures of iron status previously examined in whites were assessed in the other ethnic groups in the current study. To our knowledge, this research represents the first major assessment of candidate genetic determinants of iron deficiency and iron status measures in non-white populations. In addition to studying the association between SNPs and the primary outcome of presence of iron deficiency, a unique aspect of the statistical approach was the estimation of the effect of increasing copies of the minor allele of selected SNPs on changes in the degree of iron deficiency in multiple ethnicities.

## Methods

### Study Population

The current study utilized a subset of subjects who had been enrolled in the initial screening phase of the HEIRS Study at five Field Centers encompassing six geographic locations including Alabama, California, District of Columbia, Hawaii, and Oregon in the United States, and Ontario, Canada [17], [18]. Participants were eligible for the current study if they had not withdrawn consent and had agreed to blood storage. Selection criteria included self-report of white or Caucasian, African-American, Hispanic or Asian race/ethnicity, males at least 25 years of age and females at least 50 years. Females younger than 50 years were excluded because of pre-menopausal iron depletion from blood loss. Approval for the study was obtained from the following: Institutional Review Board of the University of California, Irvine; Institutional Review Board of the University of California, Berkeley; Institutional Review Board of the University of Minnesota; Howard University Institutional Review Board; Institutional Review Board of the University of Alabama at Birmingham; Institutional Review Board of Kaiser Permanente Northwest Center for Health Research; Institutional Review Board of Wake Forest University Health Sciences; and the University of Western Ontario Research Ethics Board for Health. Written informed consent was obtained from all participants.

Population samples consisting of cases of iron deficiency and controls were selected from the African-American, Asian, Hispanic, and white participants in the HEIRS Study. Cases of iron deficiency were defined as subjects having a serum ferritin concentration (SF) ≤12 µg/L, the point of total depletion of iron stores [19], [20]. Controls (SF >100 µg/L in men, SF >50 µg/L in women) were frequency matched 2∶1 to cases by sex and geographic location. Cases and controls who were selected from African Americans (77 cases, 144 controls), Asians (51 cases, 102 controls), and Hispanics (79 cases, 160 controls) were new to this study and had not been included in the previous GWAS. White subjects included 357 cases and 358 controls from the previous GWAS as well as additional 374 white controls added to achieve the desired 2∶1 frequency matching.

### Laboratory Methods

Laboratory methods are described in detail elsewhere [11]. Briefly, *HFE* C282Y and H63D genotypes were determined using the Invader® Assay (Third Wave Technologies, Madison WI). Lack of a detectable C282Y or H63D mutation was designated as *HFE* wild-type (wt/wt). Spectrophotometric measures of SI and UIBC levels, turbidometric immunoassay of SF (Roche Applied Science/Hitachi 911, Indianapolis, IN), and calculation of TfS were performed on non-fasting blood samples. SI, SF, UIBC, and sTfR, were analyzed using Roche reagents on the Roche/Hitachi Modular P instrument (Roche Diagnostics, Indianapolis IN). TIBC was calculated as the sum of SI + UIBC. TfS was calculated as the ratio, SI/TIBC, and expressed as a percentage. Body iron (mg/kg), an index of iron deficiency, was assessed as follows: body iron  =  -[log_10_((sTfR ×1000)/SF) −2.8229]/0.1207. In this approach, body iron is expressed as a positive value when stores are present and negatively with tissue iron deficiency [21], [22]. A body iron < −4 mg/kg body weight represents a deficit severe enough to produce anemia. However, positive values may occur in some cases of iron deficiency, for example, when sTfR is not elevated as a result of a lack of erythropoietin related to co-morbid conditions such as kidney disease. The sTfR/SF ratio was calibrated previously by quantitative phlebotomy performed in healthy subjects [23]. To exclude common environmental causes of iron deficiency, antibody testing was performed for *H. pylori*, carcinoembryonic antigen (CEA), and celiac disease. C-reactive Protein (CRP), alanine aminotransferase (ALT), and gamma-glutamyltransferase (GGT) were measured to identify acute phase protein elevations in SF.

### Sample and SNP Selection, Genotyping and Quality Control Procedures

Buffy coat DNA was extracted and purified by SDS cell lysis followed by a salt precipitation method for protein removal using commercial Puregene® reagents (Gentra System, Inc., Minneapolis, MN, now Qiagen, Valencia, CA). Using GoldenGate methodology, a custom SNP set with 1536 SNPs per array was designed to cover the number of SNPs that had been chosen for genotyping. These included 1239 SNPs as follows: a) 107 unique SNPs chosen on the basis of our previous GWAS performed in whites that showed significant associations with iron-related outcomes (p-value <0.00005), b) 67 SNPs tagging regions identified in the GWAS, c) 36 SNPs associated with iron status that had been reported in the scientific literature and that were located in *TF*, *HFE*, and *TMPRSS6* genes, among others, and d) 1029 tag SNPs located in candidate genes for iron metabolism. Additionally, 297 ancestry informative markers were genotyped to estimate the admixture proportions in the African-American and Hispanic samples (Table S1). A CEPH trio and a within-study replicate were placed on each plate to assess the concordance of genotype calls. Mendelian consistency for all trios was greater than 99% and reproducibility for the same CEPH individuals across all plates was greater than 98%. Reproducibility for the thirteen within-study replicates placed across plates was greater than 99%. Reported gender was compared to gender estimated by Illumina’s GenomeStudio, based on gender targets built into the custom OPA. Twelve samples were excluded due to a conflict between reported and genetically inferred gender. Because of allele frequency differences between the four ethnic groups, further SNP and sample quality control assessments were done separately for each group. SNPs were filtered based on call rate (<95%) and allele frequency (<0.005). Individuals were filtered on call rate (<95%), heterozygosity (>∼50%) and IBS (>90%). Quality control tests were completed using the GenABEL library [24] of the R statistical package (http://www.r-project.org/). The genotype distributions of each SNP were tested for fit to Hardy-Weinberg equilibrium HWE expectations. No SNPs were excluded from the association analysis based on the p-value of the HWE test. From the 1702 unique samples included for genotyping, there were 1084 white, 153 Asian, 212 African-American and 233 Hispanic individuals that passed the quality control assessments.

### Statistical Analyses

Eight iron-related outcomes were studied. The primary outcome was iron-deficient case-control status. Other indicators of iron status included SI, SF, TfS, sTfR, body iron, TIBC, and UIBC. With the exception of the dichotomous iron deficient case-control status variable, the variables were continuous quantitative traits. The distributions of the seven continuous outcomes and four continuous covariates were tested for their fit to the normal distribution. Natural logarithm transformations were applied to the SF, TfS and sTfR outcomes and the CEA, CRP, GGT and ALT covariates to improve their fit to the normal distribution.

Genotypes were coded as 0, 1 or 2 indicating the number of minor alleles in the genotype and were modeled as continuous variables in the multiple regression models. Covariates showing nominally significant effects on an outcome were included in multiple regression models that also included genotype effects. Odds ratios and regression coefficients were computed with odds ratios representing the multiplicative increase in risk attributed to the addition of one copy of the minor allele to the genotype (sometimes referred to as the single allele odds ratio) and regression coefficients representing the change in the outcome associated with increasing copies of the minor allele. For the African-American and Hispanic samples, ancestry proportions were estimated from ancestry informative markers (AIMs) using the STRUCTURE program with K = 2 [25]. The estimated proportion of the first ancestry component was included as a covariate in the linear and logistic regression models for the two admixed populations.

After filtering based on the quality control assessments, there were 1134, 1115, 1113 and 1134 SNPs analyzed separately for association with the eight iron-related outcomes in the white, African-American, Hispanic and Asian population samples, respectively, adjusted for covariates. Although many of the tested SNPs are correlated through linkage disequilibrium and are not independent, we applied a conservative Bonferroni correction to adjust for the multiple tests. A total of 1134 independent tests were assumed for each population sample such that the Bonferroni multiple test-corrected nominal p-value of 0.05/1134 = 4.4×10^−5^ represented the threshold for statistical significance. No further multiple test correction was made for the eight iron-related outcomes. The analysis strategy treated the four population samples as independent experiments and SNP association results obtained in one population sample were assessed for replication across the others. For a SNP that showed a statistically significant association with an iron-related outcome in a population sample (p<4.4×10^−5^), in order to be considered as evidence for replication, the remaining population samples had to show an observed p-value <0.01 for at least one of the iron-related outcomes and the direction of the effects had to be consistent with what was observed in the original sample. The statistical analysis was done using the R statistical package (http://www.r-project.org/) and the genotype association analysis used the GenABEL library of R [24].

## Results

### Iron-related Outcomes and Covariates

Table 1 shows the distribution of age, sex, the *HFE* C282Y/H63D genotype and the continuous outcomes, presented by population sample and iron deficient case-control status. Eight variables were assessed for significant covariate effects on the outcomes, including age, sex, the C282Y/H63D genotype, CagA strain infection status, and natural log of CEA, CRP, GGT, and ALT. The effect of the covariates on each outcome was assessed separately in each population sample using multiple regression models. Table 2 displays the variables that showed significant covariate effects (p-value <0.05 for regression coefficient of the variable) for each outcome and population sample. The relatively large sample size for whites compared to the other population samples could explain why more variables showed significant effects on the outcomes in that sample. The covariates shown in Table 2 were included in the multivariate models that were used to test for association between the genotypes and the outcomes in each population sample. There was a significant association between sex and the outcomes of body iron, natural log of SF, natural log of sTfR, UIBC, and TIBC, adjusted for additional covariates. Table S2 displays the observed differences in males and females with regard to the mean of the continuous iron-related outcomes, and supports the statistical adjustment for sex in the multivariate models.

**Table 1:** Descriptive statistics by population and iron deficient case-control status.

Variable^a^	White (N = 1084)		African American (N = 221)		Hispanic (N = 239)		Asian (N = 153)	
	Case	Control	Case	Control	Case	Control	Case	Control
Sample Size	357	727	77	144	79	160	51	102
								
Age (years)	59.3 (10.1)	60.9 (10.6)	57.8 (12.1)	57.1 (12.6)	56.7 (9.9)	55.8 (10.3)	54.3 (10.2)	57.0 (11.6)
Males, N (%)	85 (24%)	177 (24%)	27 (35%)	53 (37%)	19 (24%)	40 (25%)	18 (35%)	38 (37%)
HFE Genotype, N (%)								
−/−	215 (60.2%)	439 (60.4%)	74 (96.1%)	126 (87.5%)	60 (75.9%)	122 (76.3%)	50 (98.0%)	97 (95.1%)
H63D/−	90 (25.2%)	168 (23.1%)	3 (3.9%)	9 (6.3%)	14 (17.7%)	34 (21.3%)	1 (2.0%)	4 (3.9%)
C282Y/−	34 (9.5%)	75 (10.3%)	0	8 (5.6%)	3 (3.8%)	2 (1.3%)	0	1 (1.0)
H63D/H63D	9 (2.5%)	21 (2.9%)	0	0	1 (1.3%)	1 (0.6%)	0	0
C282Y/H63D	7 (2.05%)	17 (2.3%)	0	1 (0.7%)	1 (1.3%)	1 (0.6%)	0	0
C282Y/C282Y	2 (0.6%)	7 (1%)	0	0	0	0	0	0
Body Iron	−2.00 (2.45)	10.80 (2.56)	−4.30 (3.21)	10.78 (2.67)	−3.60 (3.14)	10.93 (2.72)	−3.33 (2.93)	12.3 (2.50)
SF (µg/L)	7.93 (2.34)	175.7 (146.24)	6.60 (2.51)	225.3 (213.29)	5.74 (2.19)	186.8 (310.32)	6.08 (2.15)	241.5 (146.99)
TfS (%)	13.3 (7.53)	29.6 (10.88)	9.75 (6.42)	27.6 (12.10)	11.5 (6.80)	28.1 (11.17)	10.8 (6.99)	34.9 (13.09)
sTfR (µg/L)	6.17 (3.29)	2.97 (0.96)	10.4 (6.92)	3.90 (1.45)	6.97 (4.45)	2.82 (0.98)	7.16 (4.45)	3.02 (2.23)
								
Serum Iron (µg/dL)	52.1 (27.77)	90.9 (30.21)	38.9 (24.81)	79.6 (28.83)	47.3 (27.41)	86.7 (31.11)	43.4 (27.17)	103.4 (32.17)
								
TIBC (µg/dL)	401.3 (52.19)	313.6 (44.94)	407.7 (45.60)	297.2 (43.58)	419.8 (54.97)	313.1 (40.76)	409.0 (44.81)	303.0 (37.55)
								
UIBC (µg/dL)	349.2 (62.57)	222.6 (53.20)	368.8 (54.32)	216.4 (58.63)	372.5 (62.56)	226.4 (50.58)	365.7 (54.94)	199.6 (53.13)

a Values shown are the mean (standard deviation) for quantitative variables and N (%) for qualitative variables.

**Table 2:** Covariates included in association analysis models.

Outcome	Population	Covariates^a^
Case-control	White	Age, ln(CEA), ln(GGT), ln(ALT), CagA
	African American	ln(GGT)
	Hispanic	ln(CEA)
	Asian	ln(GGT)
Body Iron	White	Age, Sex, ln(CEA), ln(GGT), C282Y/H63D
	African American	ln(GGT), CagA
	Hispanic	ln(CEA)
	Asian	Age, ln(GGT)
ln(SF)	White	Age, Sex, ln(CEA), ln(CRP), ln(GGT), C282Y/H63D
	African American	ln(GGT), CagA
	Hispanic	ln(CEA)
	Asian	ln(GGT)
ln(TfS)	White	ln(CEA), ln(CRP), ln(GGT), C282Y/H63D
	African American	ln(GGT), CagA
	Hispanic	ln(CEA), ln(ALT)
	Asian	ln(GGT)
ln(sTfR)	White	Sex, ln(CEA), C282Y/H63D
	African American	Sex
	Hispanic	ln(GGT), ln(ALT)
	Asian	Age, ln(GGT)
Serum Iron	White	ln(CEA), ln(CRP), ln(GGT), C282Y/H63D
	African American	ln(CRP), ln(GGT), CagA, C282Y/H63D
	Hispanic	ln(CEA), ln(CRP), ln(ALT)
	Asian	ln(GGT)
UIBC	White	Age, Sex, ln(CEA), ln(CRP), ln(GGT), CagA, C282Y/H63D
	African American	ln(CEA), ln(GGT), C282Y/H63D
	Hispanic	ln(CEA), ln(ALT)
	Asian	ln(GGT)
TIBC	White	Age, Sex, ln(CEA), CagA, C282Y/H63D
	African American	ln(CEA), ln(GGT)
	Hispanic	No Covariates
	Asian	Age, ln(GGT)

a Covariate definitions: *H. pylori*, carcinoembryonic antigen (CEA), c-reactive protein (CRP), alanine aminotransferase (ALT), gamma-glutamyltransferase (GGT), *HFE* gene C282Y/H63D genotype, CagA strain infection status (infected vs. non-infected).

### Associations between SNPs and Iron-related Outcomes

#### SNPs significantly associated with iron-related outcomes, after correction for multiple comparisons

Forty-nine SNPs showed statistically significant p-values, corrected for multiple testing, for at least one of the eight iron-related outcomes in at least one of the population samples (Table S3). Forty-eight of the significant associations were observed in the white population samples and one was found in the African Americans. The preponderance of significant results in the white sample was expected given that the sample size and statistical power of the sample far exceeded that of the other population samples, and because many of the SNPs were chosen based on the results of a GWAS that included 63% of the white samples analyzed in the current study. The 49 significantly associated SNPs were distributed within 14 distinct genomic regions. Eleven of the 49 associated SNPs were in the previously reported associated regions on chromosome 2p14, but not in known genes, and 20 of the 49 associated SNPs were in or around the *TF* gene on chromosome 3q22, which has been repeatedly shown to be associated with iron-related outcomes. Two SNPs on chromosome 7p21 and one SNP on 22q11 were significantly associated in the current study and were also reported in the recent GWAS. Hence, 69% of the 49 significantly associated SNPs were in regions that were reported in the recent GWAS. The remaining 15 SNPs were distributed within ten distinct genomic regions. Eight of the regions (including 10 SNPs) showed suggestive evidence for association in the previous GWAS but failed to reach genome-wide significance and hence, were not reported in that study. Four SNPs in the *TMPRSS6* gene on chromosome 22q12 showed statistically significant associations. The SNP rs10904850 in the *CUBN* gene on chromosome 10p13 was significantly associated with serum iron in the African-American sample (observed p-value  = 1.04×10^−5^), but showed no evidence for association in any of the other population samples.

#### SNPs in TF gene on chromosome 3q22

**Figure 1:**
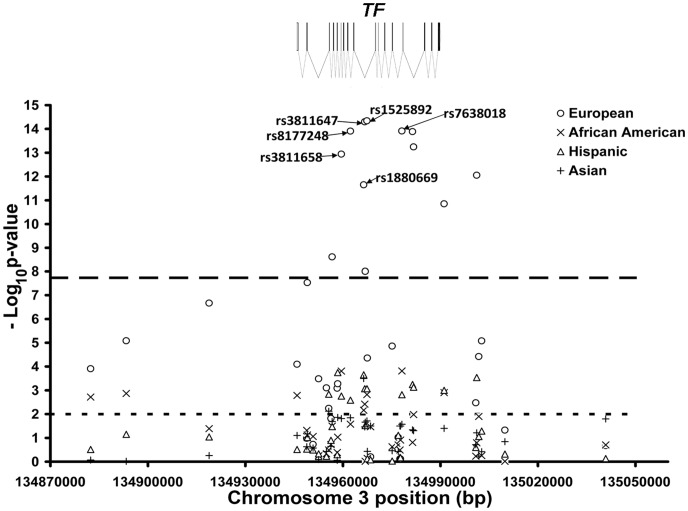
High resolution association analysis of multi-ethnic samples for chromosome 3q22. The analysis includes measured genotypes from 36 SNPs. The location and structure of the *TF* gene is shown along the top of the figure. Dashed lines indicate the threshold for statistical significance with a Bonferroni multiple test-corrected value of 4.4×10^−5^ and the threshold for replication of an association at a significance level of 0.01. The most significant associations with total iron-binding capacity (TIBC) were observed for six SNPs across the 7 kbp region (delineated) which includes exons 9, 10, and 11 of the transferrin gene, *TF*, with the measured SNP rs3811647 showing the strongest evidence for association in the white sample (O, 5.02×10^−15^) and replication at a nominal significance level of 0.01 in Hispanics (Δ, p = 0.00086), African-Americans (×, p = 0.0038), and Asians (+, p = 0.034).

SNPs in and around the *TF* gene on chromosome 3q22 showed the strongest evidence for association in the white population sample, as well as the strongest evidence for replication in the other population samples. Forty SNPs were genotyped in this region. Twenty SNPs met the multiple test corrected statistical significance threshold in the white sample for association with TIBC, 14 of which showed significant association with UIBC as well. Ten out of the 20 SNPs associated with TIBC were located within the boundaries of the *TF* gene itself. Strong evidence for association was found between TIBC and the *TF* gene SNPs in the other three population samples. Table 3 shows the association results for TIBC in the four population samples for the six *TF* SNPs that were significantly associated in the white sample and showed association in at least one other population. Although the non-white populations did not meet the multiple test corrected statistical significance threshold individually, the evidence for association within these populations is very strong given their role as replication samples. Weaker statistical evidence for association was observed between the *TF* region SNPs and the other iron-related outcomes in all samples. Figure 1 shows the –log_10_(p-values) for the 36 SNPs in the TF gene region that passed the quality control assessments and minimum allele frequency threshold in all four population samples. The location and structure of the *TF* gene is shown across the top of the figure. The most significant associations were found at rs3811647 (observed p-value  = 5.02×10^−15^) and rs1525892 (observed p-value  = 4.56×10^−15^), both located in the *TF* gene and indicated in Figure 1.

**Table 3:** Association results for six SNPs in the *TF* Gene on chromosome 3q22 and TIBC.

SNP	Position	Population Sample	Allele Frequency	Regression Parameter	P-value
rs3811658	134959542	White	0.35	20.02	1.14×10^−13^
		African American	0.17	31.86	0.00016
		Hispanic	0.45	20.15	0.0017
		Asian	0.42	20.97	0.015
rs8177248	134962316	White	0.34	20.97	1.24×10^−14^
		African American	0.12	21.51	0.027
		Hispanic	0.45	19.39	0.00261
		Asian	0.43	20.89	0.014
rs1880669	134966386	White	0.40	−18.58	2.23×10^−12^
		African American	0.65	−17.62	0.0069
		Hispanic	0.37	−23.81	0.00022
		Asian	0.51	−29.66	0.00032
rs3811647	134966719	White	0.34	21.13	5.02×10^−15^
		African American	0.23	22.32	0.0038
		Hispanic	0.46	21.45	0.00086
		Asian	0.43	18.69	0.034
rs1525892	134967402	White	0.34	21.24	4.56×10^−15^
		African American	0.27	22.97	0.00151
		Hispanic	0.46	2165	0.00086
		Asian	0.42	20.03	0.019
rs7638018	134978151	White	0.34	20.98	1.22×10^−14^
		African American	0.19	30.63	0.00015
		Hispanic	0.46	20.71	0.0015
		Asian	0.42	19.00	0.027

#### SNPs on chromosome 18q21 and in TMPRSS6 gene on chromosome 22q12

Table 4 shows the results for the SNPs on chromosomes 18q21 and 22q12 that had statistically significant associations in the white population sample and evidence for replication in at least one of the other three population samples. The SNP rs9948708 on chromosome 18q21 showed evidence for association with all the iron-related outcomes in the white sample with TIBC showing the most statistically significant association (observed p-value  = 2.9×10^−5^). Similar results were observed in the Asian population sample, where TIBC was the most significantly associated of the outcomes (observed p-value  = 0.0048) and at least nominal statistical evidence for association was observed at the majority of the outcomes. The regression parameter estimates are consistent across the white and Asian samples although the effects appear considerably stronger in the Asian sample. The larger effect size but reduced evidence of significance of the parameter estimates could be due to the lack of statistical power with the small Asian sample and differences in the minor allele frequencies; the minor allele frequency estimate for rs9948708 was 0.27 in the Asian population sample versus 0.42 in the White sample. There was no evidence for association in either the African-American or Hispanic samples.

**Table 4:** Association analysis results for SNPs on chromosomes 18q21 and 22q12 showing replication across population samples.

		White			African-American				Hispanic				Asian			
Chromosome *Gene*SNP (Position)	Outcome	Freq.^a^	Regression Parameter	P- value		Freq.^a^	Regression Parameter	P-value		Freq.^a^	Regression Parameter	P-value		Freq.^a^	Regression Parameter	P- value
Chr. 18q21	Case-Control	0.42	0.24	0.014		0.37	−0.09	0.68		0.27	0.05	0.81		0.27	0.79	0.014
rs9948708	Body Iron		−0.78	0.0053			0.18	0.82			−0.05	0.94			−2.85	0.007
(58016101)	ln(SF)		−0.13	0.0088			0.04	0.78			−0.04	0.75			−0.52	0.0048
	ln(TfS)		−0.08	0.0032			0.01	0.85			0.01	0.93			−0.20	0.066
	ln(sTfR)		0.06	0.0043			−0.02	0.71			0.01	0.94			0.14	0.079
	Serum Iron		−3.04	0.039			−2.80	0.39			1.77	0.58			−6.07	0.28
	UIBC		14.2	4.5×10^−5^			−4.39	0.63			2.70	0.75			31.05	0.017
	TIBC		11.3	2.9×10^−5^			−9.92	0.15			4.52	0.49			24.98	0.0048
Chr. 22q12	Case-Control	0.34	−0.09	0.40		0.39	−0.28	0.21		0.20	−0.28	0.30		0.31	−0.40	0.19
*TMPRSS6*	Body Iron		0.38	0.20			0.87	0.26			1.05	0.22			1.45	0.15
rs2111833	ln(SF)		0.04	0.47			0.13	0.31			0.11	0.45			0.25	0.17
(35810743)	ln(TfS)		0.11	0.00014			0.1	0.17			0.13	0.11			0.19	0.06
	ln(sTfR)		−0.05	0.029			−0.07	0.27			0.12	0.11			−0.10	0.20
	Serum Iron		7.85	4.7×10^−7^			4.57	0.17			4.74	0.21			10.43	0.044
	UIBC		−8.27	0.027			−6.17	0.51			−7.64	0.45			−32.98	0.0067
	TIBC		−0.37	0.90			−2.65	0.71			−2.01	0.80			−22.55	0.007
rs1421312	Case-Control	0.40	−0.07	0.47		0.61	0.46	0.025		0.34	−0.15	0.51		0.41	−0.16	0.56
(35817756)	Body Iron		0.1	0.15			−1.55	0.027			0.43	0.57			0.58	0.55
	ln(SF)		0.05	0.31			−0.24	0.043			0.06	0.64			0.07	0.69
	ln(TfS)		0.08	0.0018			−0.22	0.0011			0.05	0.49			0.12	0.23
	ln(sTfR)		−0.05	0.03			0.12	0.028			0.04	0.53			−0.05	0.48
	Serum Iron		6.87	3.7×10^−6^			−9.76	0.0012			1.99	0.54			4.94	0.32
	UIBC		−5.65	0.11			21.71	0.0091			0.72	0.93			−19.29	0.10
	TIBC		1.24	0.65			9.95	0.11			2.63	0.70			−14.35	0.073

a Minor allele frequency.

Two SNPs on chromosome 22q12 showed statistically significant associations in the white sample with the Asian sample providing evidence for replication at rs2111833 and the African-American sample providing replication evidence for rs1421312 (Table 4). In the white sample, rs2111833 showed the strongest associations with serum iron (observed p-value  = 4.7×10^−7^) and log-transformed transferrin saturation (observed p-value  = 0.00014). The strongest associations with rs2111833 in the Asian sample were with UIBC (observed p-value  = 0.0067) and TIBC (observed p-value  = 0.007), with weaker evidence for association with serum iron (observed p-value  = 0.044). Considerably stronger evidence for replication was seen at SNP rs1421312. Again, the most statistically significant associations in the white sample were with serum iron (observed p-value  = 3.7×10^−6^) and the log-transformed transferrin saturation (observed p-value  = 0.0018). In the African-American sample, serum iron and log-transformed transferrin saturation were the two most statistically significant associations with rs1421312, with observed p-values of 0.0012 and 0.0011, respectively. The regression coefficients for serum iron and log-transformed transferrin saturation for the white and African-American samples showed opposite signs, indicating that the direction of the effects were not consistent. However, the minor allele in the white (minor allele frequency  = 0.40) is the major allele in the Africans (minor allele frequency  = 0.61) so the opposite signs reflect associations with opposite minor alleles. If the genotypes in the African-American sample were re-coded to reflect the minor allele in the white sample then the signs of the regression coefficients would be in the same direction. No evidence for association to chromosome 22q12 was found in the Hispanic or Asian population samples.

There were more females than males in each population sample. The association analyses for SNPs on chromosomes 18q21 and 22q12 were repeated using data from females only. Although the overall sample size was smaller, the results reported in Table 5 were generally similar to those based on data from males and females (Table 4). For example, in the white sample SNPs rs2111833 and rs1421312 on *TMPRSS6* showed strong associations with serum iron with observed p-values of 5.2×10^−6^ and 7.9×10^−6^, respectively.

**Table 5:** Association analysis results of data from females only for SNPs on chromosomes 18q21 and 22q12 showing replication across population samples.

		White			African-American			Hispanic			Asian		
Chromosome *Gene*SNP (Position)	Outcome	Freq.^a^	Regression Parameter	P- value	Freq.^a^	Regression Parameter	P-value	Freq.^a^	Regression Parameter	P-value	Freq.^a^	Regression Parameter	P- value
Chr. 18q21	Case-Control	0.42	0.26	0.022	0.37	0.00	0.99	0.27	−0.04	0.85	0.27	0.54	0.15
rs9948708	Body Iron		−0.75	0.049		−0.67	0.49		−0.11	0.88		−0.53	0.67
(58016101)	ln(SF)		−0.15	0.022		−0.09	0.68		−0.04	0.83		−0.33	0.14
	ln(TfS)		−0.09	0.0027		−0.06	0.46		0.04	0.53		−0.09	0.49
	ln(sTfR)		0.06	0.016		−0.07	0.34		0.01	0.90		−0.07	0.94
	Serum Iron		−4.03	0.012		−5.05	0.18		2.17	0.51		−2.62	0.69
	UIBC		14.96	0.0001		2.38	0.85		0.67	0.94		15.71	0.37
	TIBC		11.16	0.00032		−7.14	0.42		3.53	0.63		6.60	0.56
Chr. 22q12	Case-Control	0.34	−0.09	0.47	0.39	−0.28	0.32	0.20	−0.21	0.45	0.31	−0.46	0.23
*TMPRSS6*	Body Iron		0.40	0.22		0.59	0.54		0.90	0.31		1.66	0.15
rs2111833	ln(SF)		0.05	0.45		0.15	0.49		0.12	0.54		0.22	0.32
(35810743)	ln(TfS)		0.10	0.00081		0.04	0.63		0.14	0.09		0.17	0.18
	ln(sTfR)		−0.05	0.03		−0.03	0.74		−0.13	0.44		−0.13	0.15
	Serum Iron		7.78	5.2×10^−6^		1.92	0.63		6.54	0.09		9.10	0.14
	UIBC		−7.26	0.08		2.94	0.82		−6.15	0.56		−29.38	0.077
	TIBC		0.69	0.84		0.77	0.93		1.04	0.90		−21.40	0.039
rs1421312	Case-Control	0.40	−0.05	0.67	0.61	0.54	0.024	0.34	−0.11	0.63	0.41	−0.18	0.59
(35817756)	Body Iron		0.43	0.17		−1.63	0.51		0.44	0.57		0.97	0.36
	ln(SF)		0.06	0.37		−0.36	0.05		0.07	0.67		0.02	0.93
	ln(TfS)		0.07	0.012		−0.17	0.027		0.06	0.39		0.11	0.33
	ln(sTfR)		−0.05	0.019		0.11	0.12		−0.04	0.44		−0.07	0.39
	Serum Iron		6.47	7.9×10^−6^		−6.72	0.045		3.55	0.29		6.35	0.26
	UIBC		−3.60	0.37		2.38	0.27		−0.06	0.99		−15.39	0.27
	TIBC		3.02	0.35		2.48	0.74		3.28	0.67		−12.79	0.18

a Minor allele frequency estimated from males and females.

## Discussion

The primary aim of the current study was to identify SNPs that showed association with iron status in cases with iron deficiency and control subjects across multiple ethnicities. Because patients presenting with iron deficiency are often diagnosed by using multiple measures of iron status, it is important to assess associations with each of these measures. Thus, we tested for associations not only between SNPs and a diagnosis of iron deficiency, but also with SI, TIBC, UIBC, TfS, SF, sTfR, and body iron. Three chromosomal regions showed evidence for association with one or more or these measures across the multiple populations that were sampled, including SNPs in the *TF* gene on chromosome 3q22, the *TMPRSS6* gene on chromosome 22q12, and on chromosome 18q21.

Twenty SNPs in the *TF* gene region were significantly associated with TIBC in the white sample (observed p<4.4×10^−5^). Of these, six SNPs showed replication in other ethnicities (Table 3). SNPs chosen for genotyping in the *TF* gene region that have previously shown an association with serum transferrin included rs1867504, rs4525863 and rs1830084 [15] rs3811658 and rs1880669 [26], rs1358024 and rs6794945 [15], [26], and rs3811647 [15], [26], [27]. In our study, the strongest statistical significance with TIBC, a marker for transferrin, was observed for rs3811647 in whites (observed p = 5.02×10^−15^). In our previous GWAS, this association was statistically significant (p = 7.0×10^−9^) and replicated in a sample of cases and controls selected from a population of white male and female veterans (observed p = 0.012) [11]. To our knowledge, our study is the first to replicate this observation in non-white population samples, with strongest evidence for replication in Hispanics (observed p = 0.00086).

A novel SNP rs9948708 on chromosome 18q21 satisfied the multiple-test corrected significance level for association with TIBC in the white sample (Table 4, observed p = 2.9×10^−5^), with evidence for replication in the Asian sample. To our knowledge, this SNP has not been reported in the scientific literature with regard to implications for iron deficiency. In contrast, associations between variants in the *TMPRSS6* gene and hemoglobin levels were found in individuals with Indian Asian ancestry as well as in those with European ancestry [28]. In our previous GWAS, we did not find genome-wide associations with *TMPRSS6* SNPs in the white sample. However, in this follow-up study, the number of iron-replete controls was increased and that may have increased the power of the study to detect significant associations with serum iron in the white sample with SNP rs2111833 (Table 4, observed p = 4.7×10^−7^) and SNP rs1421312 (Table 4, observed p = 3.7×10^−6^), with evidence for replication in Asians and African-Americans, respectively.

Of the 49 SNPs that were statistically significantly associated with at least one iron-related outcome (observed p<4.4×10^−5^), only one SNP showed a statistically significant association at the multiple test-corrected significance level in a non-white sample. SNP rs10904850 in the *CUBN* gene on chromosome 10p13 was significantly associated with serum iron in the African-American sample (observed p = 1.0×10^−5^), but no evidence for association with this SNP was observed in any of the three other samples. The minor allele frequency was 0.13 in African Americans, compared with 0.33 in whites, 0.17 in Hispanics, and 0.19 in Asians. The *CUBN* gene plays a role in vitamin and iron metabolism by facilitating their uptake. Cubilin and megalin are responsible for reabsorbing transferrin iron in the kidney urine collecting system [29] and the influence of *CUBN* on chronic kidney disease in African Americans has been studied [30]. We found that African-American controls had the lowest iron and TIBC concentrations of all the groups (Table 1). A possible explanation is that the cubilin SNP is affecting serum iron through an influence on efficiency of reabsorption of iron-bearing transferrin in the kidney.

In this study, four population samples were examined as a follow-up to a genome wide association study of iron deficiency conducted in white participants enrolled in the HEIRS Study. An advantage of the research design was the genotyping of a panel of markers that show large frequency differentials between major geographic ancestral groupings [31], [32] and this enabled estimation of ancestry proportions for African-American and Hispanic samples. Additionally, models incorporated information from relevant demographic and clinical measures to control for the effects including environmental causes of iron deficiency. Observed similarities and differences in age, sex, iron measures and the distribution of *HFE* genotypes are shown in Table 1. Although frequency matched by sex and geographic location, on average, whites were three years older than the African-American sample and 4.3 years older than Hispanics and Asians. The proportion of whites with *HFE* gene mutations was higher than that in the other groups; there were nine C282Y homozygotes (two cases and seven controls) compare to zero in the non-white samples. Generally, females had lower observed mean values for iron-related outcomes than males, especially within the controls (Table S2). This provides further support to the inclusion of sex as a covariate in multiple regression models when there was a significant association between sex and the outcome. A limitation to the study was the relatively small sizes of the non-white population samples, thus lack of association between some SNPs and iron measures may have been due to low statistical power.

In summary, SNPs were identified that showed association across the multiple populations that were sampled. We found a novel SNP in *TMPRSS6* that was associated with serum iron in whites and replicated in African Americans, suggesting a role for this SNP in increasing the risk of iron deficiency in affected persons. Our results confirm known associations between SNPs in the *TF* and *TMPRSS6* genes with TIBC and UIBC and give evidence of their role in different ethnic groups, a unique aspect of this study, and suggest the possibility of origins in a common founder.

## Supporting Information

Table S1 **Basis of SNP selection for genotyping.** a) SNPs significantly associated with iron-related outcomes in a previous GWAS performed in whites (GWAS, n = 107), b) SNPs tagging regions identified in the GWAS (GWAS region, n = 67), c) SNPs associated with iron status and reported in the scientific literature (Literature, n = 36),d) tag SNPs located in candidate genes for iron metabolism (Gene, n = 1029), and e) ancestry informative marker (AIM, n = 297).
(DOCX)

Table S2 **Descriptive statistics by population, iron deficient case-control status, and sex.**
(DOC)

Table S3 **Forty-nine SNPs that showed statistically significant p-values, corrected for multiple testing, for at least one of eight iron-related outcomes in the white sample (48 SNPS) or the African-American sample (one SNP).**
(DOCX)
