# Supplementary material for: Associations between Single Nucleotide Polymorphisms in Iron-Related Genes and Iron Status in Multiethnic Populations
Source: PLoS One. 2012 Jun 22;7(6):e38339. doi: 10.1371/journal.pone.0038339 (PMC3382217; doi:10.1371/journal.pone.0038339)
Supplement: Table S1 — Basis of SNP selection for genotyping. a) SNPs significantly associated with iron-related outcomes in a previous GWAS performed in whites (GWAS, n = 107), b) SNPs tagging regions identified in the GWAS (GWAS region, n = 67), c) SNPs associated with iron status and reported in the scientific literature (Literature, n = 36),d) tag SNPs located in candidate genes for iron metabolism (Gene, n = 1029), and e) ancestry informative marker (AIM, n = 297). (DOCX) [file pone.0038339.s001.docx]

Table S1. Basis of SNP selection for genotyping: a) SNPs significantly associated with iron-related outcomes in a previous GWAS performed in whites (GWAS, n=107), b) SNPs tagging regions identified in the GWAS (GWAS tag SNP, n=67), c) SNPs associated with iron status and reported in the scientific literature (Literature, n=36),d) tag SNPs located in candidate genes for iron metabolism (Gene, n=1029), and e) ancestry informative marker (AIM, n=297).

| **SNP Name** | | **Chr** | **Position** | **Gene** | **Basis for SNP selection** |
| --- | --- | --- | --- | --- | --- |
| rs884080 | | 1 | 2058911 |  | AIM |
| rs7528979 | | 1 | 10027723 |  | AIM |
| rs2273348 | | 1 | 11013343 |  | AIM |
| rs4846012 | | 1 | 11492192 |  | AIM |
| rs2817611 | | 1 | 11535750 |  | AIM |
| rs766325 | | 1 | 18701764 |  | AIM |
| rs1466286 | | 1 | 26554872 |  | AIM |
| rs204057 | | 1 | 29092673 |  | AIM |
| rs2007350 | | 1 | 29466743 |  | AIM |
| rs6684063 | | 1 | 30471927 |  | AIM |
| rs2016160 | | 1 | 47283621 |  | AIM |
| rs1934393 | | 1 | 48981205 |  | AIM |
| rs3768176 | | 1 | 57340593 |  | AIM |
| rs1501225 | | 1 | 60610101 |  | AIM |
| rs1739897 | | 1 | 75828083 |  | AIM |
| rs2103119 | | 1 | 79970160 |  | GWAS |
| rs1536570 | | 1 | 81417598 |  | AIM |
| rs3828121 | | 1 | 82194788 |  | AIM |
| rs11163801 | | 1 | 83922387 |  | GWAS |
| rs926685 | | 1 | 84040000 |  | GWAS |
| rs1229133 | | 1 | 118706429 |  | AIM |
| rs16827043 | | 1 | 144106797 |  | Literature |
| rs10218795 | | 1 | 144127413 | *HFE2* | Gene |
| rs1535921 | | 1 | 144129407 | *HFE2* | Gene |
| rs3806218 | | 1 | 145478407 |  | AIM |
| rs3767627 | | 1 | 148205522 |  | GWAS |
| rs952146 | | 1 | 152635552 | *IL6R* | Gene |
| rs17654071 | | 1 | 152635876 | *IL6R* | Gene |
| rs2054855 | | 1 | 152637562 | *IL6R* | Gene |
| rs6427641 | | 1 | 152647110 | *IL6R* | Gene |
| rs1386821 | | 1 | 152648673 | *IL6R* | Gene |
| rs12090237 | | 1 | 152656365 | *IL6R* | Gene |
| rs6684439 | | 1 | 152662463 | *IL6R* | Gene |
| rs4845618 | | 1 | 152666639 | *IL6R* | Gene |
| rs6427658 | | 1 | 152667423 | *IL6R* | Gene |
| rs7549250 | | 1 | 152670960 | *IL6R* | Gene |
| rs7518199 | | 1 | 152674043 | *IL6R* | Gene |
| rs4845371 | | 1 | 152674964 | *IL6R* | Gene |
| rs4553185 | | 1 | 152677579 | *IL6R* | Gene |
| rs4537545 | | 1 | 152685503 | *IL6R* | Gene |
| rs4845626 | | 1 | 152690109 | *IL6R* | Gene |
| rs4129267 | | 1 | 152692888 | *IL6R* | Gene |
| rs11265618 | | 1 | 152696716 | *IL6R* | Gene |
| rs4329505 | | 1 | 152699044 | *IL6R* | Gene |
| rs4240872 | | 1 | 152702819 | *IL6R* | Gene |
| rs2229238 | | 1 | 152704520 | *IL6R* | Gene |
| rs4072391 | | 1 | 152705504 | *IL6R* | Gene |
| rs7526293 | | 1 | 152710833 | *IL6R* | Gene |
| rs915180 | | 1 | 152892156 |  | AIM |
| rs2592888 | | 1 | 157852197 |  | AIM |
| rs1027702 | | 1 | 158444515 |  | AIM |
| rs3737792 | | 1 | 158781890 |  | GWAS |
| rs164176 | | 1 | 160622113 |  | GWAS |
| rs164123 | | 1 | 160634278 |  | GWAS |
| rs2632549 | | 1 | 160638494 |  | GWAS |
| rs351453 | | 1 | 160642540 |  | GWAS |
| rs4657449 | | 1 | 163731905 |  | AIM |
| rs10753801 | | 1 | 168526468 |  | GWAS |
| rs1409778 | | 1 | 196044182 |  | AIM |
| rs2644548 | | 1 | 211088985 | *FLVCR1* | Gene |
| rs1874420 | | 1 | 211108726 | *FLVCR1* | Gene |
| rs2291773 | | 1 | 211127653 | *FLVCR1* | Gene |
| rs1390501 | | 1 | 211131668 | *FLVCR1* | Gene |
| rs3207090 | | 1 | 211135218 | *FLVCR1* | Gene |
| rs6679775 | | 1 | 211135608 | *FLVCR1* | Gene |
| rs1284852 | | 1 | 211139636 | *FLVCR1* | Gene |
| rs1284851 | | 1 | 211139989 | *FLVCR1* | Gene |
| rs1043641 | | 1 | 224399616 |  | Literature |
| rs7535375 | | 1 | 233979782 |  | AIM |
| rs1339737 | | 1 | 235717811 |  | AIM |
| rs2291409 | | 1 | 240058238 |  | AIM |
| rs2290753 | | 1 | 240132479 |  | AIM |
| rs6426327 | | 1 | 242415852 |  | AIM |
| rs2642995 | | 1 | 243498606 |  | AIM |
| rs813779 | | 2 | 5540855 |  | AIM |
| rs2001660 | | 2 | 9564039 |  | AIM |
| rs1510834 | | 2 | 13694789 |  | AIM |
| rs6741005 | | 2 | 16031174 |  | GWAS tag SNP |
| rs16855322 | | 2 | 16031746 |  | GWAS tag SNP |
| rs6431746 | | 2 | 16032654 |  | GWAS tag SNP |
| rs11695713 | | 2 | 16033209 |  | GWAS tag SNP |
| rs10803676 | | 2 | 16035958 |  | GWAS tag SNP |
| rs11685620 | | 2 | 16039315 |  | GWAS tag SNP |
| rs11903230 | | 2 | 16040473 |  | GWAS tag SNP |
| rs6735681 | | 2 | 16040624 |  | GWAS |
| rs6751458 | | 2 | 16046274 |  | GWAS tag SNP |
| rs520354 | | 2 | 21171264 |  | AIM |
| rs1073319 | | 2 | 29293958 |  | AIM |
| rs163077 | | 2 | 38197256 |  | AIM |
| rs1470524 | | 2 | 44983019 |  | AIM |
| rs6739083 | | 2 | 46371633 | *EPAS1* | Gene |
| rs1867786 | | 2 | 46372398 | *EPAS1* | Gene |
| rs10197384 | | 2 | 46373888 | *EPAS1* | Gene |
| rs13428739 | | 2 | 46377438 | *EPAS1* | Gene |
| rs1867782 | | 2 | 46381044 | *EPAS1* | Gene |
| rs10166198 | | 2 | 46384562 | *EPAS1* | Gene |
| rs11894252 | | 2 | 46386880 | *EPAS1* | Gene |
| rs2121266 | | 2 | 46389428 | *EPAS1* | Gene |
| rs17034950 | | 2 | 46392298 | *EPAS1* | Gene |
| rs11689011 | | 2 | 46394680 | *EPAS1* | Gene |
| rs7571879 | | 2 | 46395912 | *EPAS1* | Gene |
| rs2044456 | | 2 | 46399820 | *EPAS1* | Gene |
| rs6720535 | | 2 | 46400998 | *EPAS1* | Gene |
| rs9973653 | | 2 | 46401613 | *EPAS1* | Gene |
| rs6753302 | | 2 | 46402410 | *EPAS1* | Gene |
| rs7582701 | | 2 | 46404273 | *EPAS1* | Gene |
| rs4953342 | | 2 | 46405551 | *EPAS1* | Gene |
| rs4953344 | | 2 | 46405962 | *EPAS1* | Gene |
| rs4953345 | | 2 | 46406105 | *EPAS1* | Gene |
| rs4952820 | | 2 | 46407470 | *EPAS1* | Gene |
| rs1868089 | | 2 | 46408150 | *EPAS1* | Gene |
| rs13419896 | | 2 | 46409849 | *EPAS1* | Gene |
| rs9679290 | | 2 | 46411148 | *EPAS1* | Gene |
| rs4953347 | | 2 | 46411729 | *EPAS1* | Gene |
| rs6758592 | | 2 | 46412045 | *EPAS1* | Gene |
| rs12617313 | | 2 | 46413280 | *EPAS1* | Gene |
| rs6726454 | | 2 | 46414706 | *EPAS1* | Gene |
| rs6706003 | | 2 | 46416896 | *EPAS1* | Gene |
| rs4953352 | | 2 | 46419026 | *EPAS1* | Gene |
| rs4953353 | | 2 | 46420780 | *EPAS1* | Gene |
| rs2346175 | | 2 | 46424814 | *EPAS1* | Gene |
| rs10191091 | | 2 | 46426714 | *EPAS1* | Gene |
| rs10199201 | | 2 | 46430666 | *EPAS1* | Gene |
| rs6756667 | | 2 | 46432913 | *EPAS1* | Gene |
| rs1868087 | | 2 | 46433223 | *EPAS1* | Gene |
| rs7589621 | | 2 | 46435886 | *EPAS1* | Gene |
| rs6743991 | | 2 | 46436739 | *EPAS1* | Gene |
| rs6707241 | | 2 | 46438356 | *EPAS1* | Gene |
| rs1374748 | | 2 | 46440538 | *EPAS1* | Gene |
| rs7583554 | | 2 | 46440601 | *EPAS1* | Gene |
| rs6755594 | | 2 | 46442799 | *EPAS1* | Gene |
| rs3768728 | | 2 | 46444295 | *EPAS1* | Gene |
| rs3768729 | | 2 | 46445510 | *EPAS1* | Gene |
| rs3768730 | | 2 | 46446028 | *EPAS1* | Gene |
| rs2346176 | | 2 | 46446803 | *EPAS1* | Gene |
| rs3754556 | | 2 | 46448994 | *EPAS1* | Gene |
| rs1374749 | | 2 | 46449937 | *EPAS1* | Gene |
| rs1992846 | | 2 | 46451085 | *EPAS1* | Gene |
| rs4953361 | | 2 | 46452072 | *EPAS1* | Gene |
| rs7594912 | | 2 | 46452877 | *EPAS1* | Gene |
| rs3088359 | | 2 | 46455755 | *EPAS1* | Gene |
| rs7583088 | | 2 | 46456669 | *EPAS1* | Gene |
| rs7594278 | | 2 | 46458097 | *EPAS1* | Gene |
| rs7571218 | | 2 | 46459163 | *EPAS1* | Gene |
| rs13019268 | | 2 | 46463716 | *EPAS1* | Gene |
| rs7577700 | | 2 | 46464109 | *EPAS1* | Gene |
| rs1868092 | | 2 | 46467706 | *EPAS1* | Gene |
| rs13424253 | | 2 | 46469569 | *EPAS1* | Gene |
| rs11689694 | | 2 | 46470686 | *EPAS1* | Gene |
| rs842634 | | 2 | 60944726 |  | AIM |
| rs1816618 | | 2 | 64301388 |  | GWAS |
| rs10496110 | | 2 | 64303859 |  | GWAS tag SNP |
| rs1406576 | | 2 | 64306614 |  | GWAS tag SNP |
| rs2555435 | | 2 | 64306990 |  | GWAS tag SNP |
| rs17738968 | | 2 | 64308705 |  | GWAS tag SNP |
| rs871776 | | 2 | 64309178 |  | GWAS |
| rs2698531 | | 2 | 64310892 |  | GWAS tag SNP |
| rs1019684 | | 2 | 64314481 |  | GWAS tag SNP |
| rs10496111 | | 2 | 64315987 |  | GWAS tag SNP |
| rs2698524 | | 2 | 64324864 |  | GWAS tag SNP |
| rs13027331 | | 2 | 64325209 |  | GWAS tag SNP |
| rs6750096 | | 2 | 64327573 |  | GWAS |
| rs2303496 | | 2 | 64330033 |  | GWAS tag SNP |
| rs1025209 | | 2 | 64332564 |  | GWAS |
| rs13015676 | | 2 | 64338633 |  | GWAS tag SNP |
| rs2251764 | | 2 | 64338969 |  | GWAS tag SNP |
| rs2114432 | | 2 | 64339316 |  | GWAS tag SNP |
| rs2698541 | | 2 | 64342183 |  | GWAS |
| rs2555442 | | 2 | 64342795 |  | GWAS tag SNP |
| rs890482 | | 2 | 64346439 |  | GWAS tag SNP |
| rs869738 | | 2 | 64346926 |  | GWAS tag SNP |
| rs2433388 | | 2 | 64347123 |  | GWAS tag SNP |
| rs17739675 | | 2 | 64348417 |  | GWAS tag SNP |
| rs1529102 | | 2 | 64349131 |  | GWAS tag SNP |
| rs2698527 | | 2 | 64353133 |  | GWAS tag SNP |
| rs1426710 | | 2 | 64355179 |  | GWAS tag SNP |
| rs2698530 | | 2 | 64357399 |  | GWAS |
| rs2698538 | | 2 | 64361246 |  | GWAS tag SNP |
| rs10200259 | | 2 | 64363303 |  | GWAS tag SNP |
| rs2433384 | | 2 | 64363623 |  | GWAS tag SNP |
| rs7600002 | | 2 | 64366711 |  | GWAS tag SNP |
| rs2008312 | | 2 | 65366295 |  | AIM |
| rs1457451 | | 2 | 65715882 |  | Literature |
| rs736779 | | 2 | 68483570 |  | AIM |
| rs1986601 | | 2 | 70119154 |  | AIM |
| rs975612 | | 2 | 72300989 |  | AIM |
| rs828869 | | 2 | 74235155 |  | AIM |
| rs10515919 | | 2 | 75394104 |  | AIM |
| rs1427648 | | 2 | 82432439 |  | GWAS |
| rs1469369 | | 2 | 82605321 |  | GWAS |
| rs4852696 | | 2 | 83005152 |  | AIM |
| rs1019837 | | 2 | 83326738 |  | AIM |
| rs1015117 | | 2 | 86652521 |  | AIM |
| rs1796048 | | 2 | 97065450 |  | AIM |
| rs3860446 | | 2 | 103855783 |  | AIM |
| rs2376070 | | 2 | 104012846 |  | GWAS |
| rs7605935 | | 2 | 109338839 |  | GWAS |
| rs11898400 | | 2 | 109340493 |  | GWAS |
| rs6749211 | | 2 | 109342097 |  | GWAS |
| rs7592947 | | 2 | 113239601 | *IL1A* | Gene |
| rs17561 | | 2 | 113253694 | *IL1A* | Gene |
| rs3783525 | | 2 | 113258290 | *IL1A* | Gene |
| rs2856837 | | 2 | 113258396 | *IL1A* | Gene |
| rs1800587 | | 2 | 113259431 | *IL1A* | Gene |
| rs3783516 | | 2 | 113262149 | *IL1A* | Gene |
| rs3917368 | | 2 | 113299253 | *IL1B* | Gene |
| rs1143634 | | 2 | 113306861 | *IL1B* | Gene |
| rs1143633 | | 2 | 113306938 | *IL1B* | Gene |
| rs3136558 | | 2 | 113307746 | *IL1B* | Gene |
| rs1143627 | | 2 | 113310858 | *IL1B* | Gene |
| rs16944 | | 2 | 113311338 | *IL1B* | Gene |
| rs12621220 | | 2 | 113314726 | *IL1B* | Gene |
| rs1446125 | | 2 | 119687945 | *STEAP3* | Gene |
| rs11677262 | | 2 | 119688615 | *STEAP3* | Gene |
| rs7595954 | | 2 | 119691991 | *STEAP3* | Gene |
| rs895397 | | 2 | 119693725 | *STEAP3* | Gene |
| rs838065 | | 2 | 119693801 | *STEAP3* | Gene |
| rs865688 | | 2 | 119699720 | *STEAP3* | Gene |
| rs838102 | | 2 | 119701203 | *STEAP3* | Gene |
| rs3769659 | | 2 | 119702453 | *STEAP3* | Gene |
| rs865108 | | 2 | 119702854 | *STEAP3* | Gene |
| rs708670 | | 2 | 119703707 | *STEAP3* | Gene |
| rs708672 | | 2 | 119703824 | *STEAP3* | Gene |
| rs838100 | | 2 | 119705035 | *STEAP3* | Gene |
| rs838095 | | 2 | 119706106 | *STEAP3* | Gene |
| rs838092 | | 2 | 119712623 | *STEAP3* | Gene |
| rs838090 | | 2 | 119713629 | *STEAP3* | Gene |
| rs838086 | | 2 | 119717972 | *STEAP3* | Gene |
| rs708675 | | 2 | 119725827 | *STEAP3* | Gene |
| rs12711924 | | 2 | 119728641 | *STEAP3* | Gene |
| rs1867749 | | 2 | 119730382 | *STEAP3* | Gene |
| rs838073 | | 2 | 119731079 | *STEAP3* | Gene |
| rs6720040 | | 2 | 119736001 | *STEAP3* | Gene |
| rs3731603 | | 2 | 119738387 | *STEAP3* | Gene |
| rs11694139 | | 2 | 119739730 | *STEAP3* | Gene |
| rs1530561 | | 2 | 119740912 | *STEAP3* | Gene |
| rs6721852 | | 2 | 119740973 | *STEAP3* | Gene |
| rs1036543 | | 2 | 133392684 |  | AIM |
| rs7591869 | | 2 | 140376310 |  | Literature |
| rs2711070 | | 2 | 159210779 |  | AIM |
| rs7591849 | | 2 | 159821127 |  | GWAS |
| rs964176 | | 2 | 160149391 |  | AIM |
| rs1521527 | | 2 | 165253332 |  | AIM |
| rs13413169 | | 2 | 167516694 |  | GWAS |
| rs10490603 | | 2 | 172077646 | *CYBRD1* | Gene |
| rs17221848 | | 2 | 172080839 | *CYBRD1* | Gene |
| rs1476110 | | 2 | 172081753 | *CYBRD1* | Gene |
| rs13406777 | | 2 | 172082641 | *CYBRD1* | Gene |
| rs11680268 | | 2 | 172083703 | *CYBRD1* | Gene |
| rs3806562 | | 2 | 172085031 | *CYBRD1* | Gene |
| rs884409 | | 2 | 172086903 |  | Literature |
| rs960748 | | 2 | 172088182 | *CYBRD1* | Gene |
| rs6759240 | | 2 | 172089044 | *CYBRD1* | Gene |
| rs6734372 | | 2 | 172097050 | *CYBRD1* | Gene |
| rs12476341 | | 2 | 172105923 | *CYBRD1* | Gene |
| rs7586144 | | 2 | 172106557 | *CYBRD1* | Gene |
| rs13009270 | | 2 | 172110783 | *CYBRD1* | Gene |
| rs7596578 | | 2 | 172110913 | *CYBRD1* | Gene |
| rs17554 | | 2 | 172111615 | *CYBRD1* | Gene |
| rs11684782 | | 2 | 172114235 | *CYBRD1* | Gene |
| rs2542941 | | 2 | 172117967 | *CYBRD1* | Gene |
| rs10455 | | 2 | 172119519 | *CYBRD1* | Gene and Literature |
| rs950163 | | 2 | 172119983 | *CYBRD1* | Gene |
| rs2542940 | | 2 | 172120756 | *CYBRD1* | Gene |
| rs7585194 | | 2 | 172120862 | *CYBRD1* | Gene |
| rs3821083 | | 2 | 172121153 | *CYBRD1* | Gene |
| rs2674485 | | 2 | 172122918 | *CYBRD1* | Gene |
| rs10490602 | | 2 | 172127511 | *CYBRD1* | Gene |
| rs868179 | | 2 | 177257743 |  | AIM |
| rs12693541 | | 2 | 190126935 | *SLC40A1* | Gene |
| rs2352262 | | 2 | 190132030 | *SLC40A1* | Gene |
| rs2304704 | | 2 | 190138422 | *SLC40A1* | Gene and Literature |
| rs4667287 | | 2 | 190140120 | *SLC40A1* | Gene |
| rs994227 | | 2 | 190140712 | *SLC40A1* | Gene |
| rs1439814 | | 2 | 190151138 | *SLC40A1* | Gene |
| rs1123109 | | 2 | 190152637 | *SLC40A1* | Gene |
| rs1439816 | | 2 | 190152875 | *SLC40A1* | Gene |
| rs10202029 | | 2 | 190154529 | *SLC40A1* | Gene |
| rs17198983 | | 2 | 190156056 | *SLC40A1* | Gene |
| rs2352267 | | 2 | 190157210 | *SLC40A1* | Gene |
| rs10497705 | | 2 | 190200259 |  | AIM |
| rs9288362 | | 2 | 205829029 |  | GWAS |
| rs1155683 | | 2 | 212560914 |  | GWAS |
| rs2054615 | | 2 | 213310258 |  | AIM |
| rs1554622 | | 2 | 219431723 |  | AIM |
| rs1517634 | | 2 | 223891729 |  | AIM |
| rs10498255 | | 2 | 231320474 |  | AIM |
| rs883434 | | 2 | 233040183 |  | AIM |
| rs4675966 | | 2 | 241964332 |  | AIM |
| rs304051 | | 3 | 4553306 |  | AIM |
| rs749477 | | 3 | 10631823 |  | AIM |
| rs729639 | | 3 | 13801855 |  | AIM |
| rs1374197 | | 3 | 17369619 |  | AIM |
| rs1498991 | | 3 | 20875136 |  | AIM |
| rs9310888 | | 3 | 29261766 |  | AIM |
| rs1351631 | | 3 | 43493171 |  | AIM |
| rs737516 | | 3 | 43533089 |  | AIM |
| rs1013758 | | 3 | 43601379 |  | AIM |
| rs2234358 | | 3 | 45964048 |  | GWAS |
| rs2352984 | | 3 | 49923732 | *MON1A* | Gene |
| rs868891 | | 3 | 49924075 | *MON1A* | Gene |
| rs6771546 | | 3 | 49943576 | *MON1A* | Gene |
| rs7613875 | | 3 | 49946518 | *MON1A* | Gene |
| rs893367 | | 3 | 53884771 |  | AIM |
| rs1392702 | | 3 | 56809019 |  | AIM |
| rs10510791 | | 3 | 57269125 |  | AIM |
| rs1996818 | | 3 | 70395474 |  | AIM |
| rs1441443 | | 3 | 74005900 |  | AIM |
| rs11128045 | | 3 | 88580724 |  | GWAS |
| rs771767 | | 3 | 103231328 |  | GWAS |
| rs1039524 | | 3 | 115494964 |  | AIM |
| rs1147696 | | 3 | 121602169 |  | AIM |
| rs1919550 | | 3 | 122846863 |  | AIM |
| rs9740 | | 3 | 123487743 |  | AIM |
| rs2370409 | | 3 | 134492512 |  | AIM |
| rs2718812 | | 3 | 134882392 |  | Literature |
| rs1867504 | | 3 | 134893351 |  | Literature |
| rs4525863 | | 3 | 134918826 |  | GWAS and Literature |
| rs8177177 | | 3 | 134945885 | *TF* | Gene |
| rs1800277 | | 3 | 134948845 | *TF* | Gene |
| rs6796795 | | 3 | 134948912 | *TF* | Gene |
| rs4428180 | | 3 | 134949064 | *TF* | Gene |
| rs8177191 | | 3 | 134950829 | *TF* | Gene |
| rs8177201 | | 3 | 134952545 | *TF* | Gene |
| rs8177213 | | 3 | 134954917 | *TF* | Gene |
| rs8177215 | | 3 | 134955648 | *TF* | Gene |
| rs8177220 | | 3 | 134956315 | *TF* | Gene |
| rs8177224 | | 3 | 134956693 | *TF* | Gene |
| rs4241357 | | 3 | 134958253 | *TF* | Gene |
| rs1799852 | | 3 | 134958412 | *TF* | Gene and Literature |
| rs1799899 | | 3 | 134958502 |  | Literature |
| rs3811658 | | 3 | 134959542 | *TF* | Gene |
| rs2718796 | | 3 | 134961890 | *TF* | Gene |
| rs8177248 | | 3 | 134962316 | *TF* | Gene |
| rs2715631 | | 3 | 134965579 |  | GWAS tag SNP |
| rs1880669 | | 3 | 134966386 | *TF* | Gene |
| rs3811647 | | 3 | 134966719 | *TF* | GWAS, Gene and Literature |
| rs1358024 | | 3 | 134966878 |  | GWAS and Literature |
| rs1525892 | | 3 | 134967402 | *TF* | Gene |
| rs8177277 | | 3 | 134967520 |  | GWAS tag SNP |
| rs2692695 | | 3 | 134968144 |  | GWAS tag SNP |
| rs2715632 | | 3 | 134968520 | *TF* | Gene |
| rs8177333 | | 3 | 134972199 |  | GWAS tag SNP |
| rs9824452 | | 3 | 134975161 |  | GWAS tag SNP |
| rs1049296 | | 3 | 134977044 | *TF* | GWAS and Literature |
| rs2715627 | | 3 | 134977539 | *TF* | Gene |
| rs1115219 | | 3 | 134977707 | *TF* | Gene |
| rs7638018 | | 3 | 134978151 | *TF* | Gene |
| rs4854760 | | 3 | 134981431 | *TF* | Gene |
| rs4854762 | | 3 | 134981753 |  | GWAS tag SNP |
| rs9843728 | | 3 | 134984097 | *TF* | Gene |
| rs1830084 | | 3 | 134991154 |  | GWAS tag SNP |
| rs6794676 | | 3 | 135000689 |  | GWAS tag SNP |
| rs9881405 | | 3 | 135000888 |  | GWAS tag SNP |
| rs6794945 | | 3 | 135001153 |  | GWAS and Literature |
| rs13061203 | | 3 | 135001825 |  | GWAS tag SNP |
| rs9853615 | | 3 | 135002671 |  | GWAS tag SNP |
| rs7650925 | | 3 | 135009859 |  | GWAS tag SNP |
| rs2280673 | | 3 | 135040844 |  | Literature |
| rs531577 | | 3 | 139883997 |  | AIM |
| rs7636389 | | 3 | 150363816 | *CP* | Gene |
| rs3732557 | | 3 | 150364363 | *CP* | Gene |
| rs2681092 | | 3 | 150367767 | *CP* | Gene |
| rs16861579 | | 3 | 150377816 | *CP* | Gene |
| rs16861582 | | 3 | 150379120 | *CP* | Gene |
| rs13095262 | | 3 | 150397852 | *CP* | Gene |
| rs772908 | | 3 | 150406346 | *CP* | Gene |
| rs3816893 | | 3 | 150410401 | *CP* | Gene |
| rs16861634 | | 3 | 150414214 | *CP* | Gene |
| rs11709714 | | 3 | 150414670 | *CP* | Gene |
| rs7652826 | | 3 | 150421640 | *CP* | Gene |
| rs17838831 | | 3 | 150422551 | *CP* | Gene |
| rs701748 | | 3 | 150423965 | *CP* | Gene |
| rs3755641 | | 3 | 150424588 | *CP* | Gene |
| rs11708215 | | 3 | 150425954 | *CP* | Gene |
| rs1984473 | | 3 | 157293978 |  | AIM |
| rs1472578 | | 3 | 160291340 |  | AIM |
| rs1468924 | | 3 | 180465671 |  | AIM |
| rs6808013 | | 3 | 187432931 |  | AIM |
| rs1075870 | | 3 | 196162579 |  | AIM |
| rs6583286 | | 3 | 197252582 | *TFRC* | Gene |
| rs570 | | 3 | 197260637 | *TFRC* | Gene |
| rs406271 | | 3 | 197261373 | *TFRC* | Gene |
| rs17788379 | | 3 | 197261608 | *TFRC* | Gene |
| rs1805051 | | 3 | 197263369 |  | Literature |
| rs3326 | | 3 | 197265903 | *TFRC* | Gene |
| rs507131 | | 3 | 197273825 | *TFRC* | Gene |
| rs2300774 | | 3 | 197278109 | *TFRC* | Gene |
| rs3817672 | | 3 | 197285208 | *TFRC* | Gene and Literature |
| rs2686085 | | 3 | 198711008 |  | AIM |
| rs726111 | | 4 | 6030055 |  | AIM |
| rs1398829 | | 4 | 21632373 |  | AIM |
| rs6824707 | | 4 | 52405942 |  | GWAS |
| rs6818147 | | 4 | 52442331 |  | GWAS |
| rs1519590 | | 4 | 52541357 |  | GWAS |
| rs999634 | | 4 | 52768041 |  | AIM |
| rs10517518 | | 4 | 61478011 |  | AIM |
| rs717239 | | 4 | 77532922 |  | AIM |
| rs1383972 | | 4 | 86741508 |  | AIM |
| rs10516969 | | 4 | 96529186 |  | Literature |
| rs10516970 | | 4 | 96529259 |  | Literature |
| rs1491233 | | 4 | 100833238 |  | AIM |
| rs1551740 | | 4 | 115734909 |  | AIM |
| rs1459531 | | 4 | 119100475 |  | AIM |
| rs1880863 | | 4 | 123239547 |  | AIM |
| rs4975220 | | 4 | 129414784 | *PGRMC2* | Gene |
| rs4975180 | | 4 | 129417852 | *PGRMC2* | Gene |
| rs7677888 | | 4 | 129419969 | *PGRMC2* | Gene |
| rs9307613 | | 4 | 130576854 |  | AIM |
| rs1017507 | | 4 | 135500593 |  | AIM |
| rs6848090 | | 4 | 146616136 | *SMAD1* | Gene |
| rs6537355 | | 4 | 146622042 | *SMAD1* | Gene |
| rs17020201 | | 4 | 146624603 | *SMAD1* | Gene |
| rs11944685 | | 4 | 146633717 | *SMAD1* | Gene |
| rs2068991 | | 4 | 146642965 | *SMAD1* | Gene |
| rs2118438 | | 4 | 146647834 | *SMAD1* | Gene |
| rs7661162 | | 4 | 146650604 | *SMAD1* | Gene |
| rs7662541 | | 4 | 146654130 | *SMAD1* | Gene |
| rs714195 | | 4 | 146665130 | *SMAD1* | Gene |
| rs2043779 | | 4 | 146667239 | *SMAD1* | Gene |
| rs11100883 | | 4 | 146670420 | *SMAD1* | Gene |
| rs6852200 | | 4 | 146672223 | *SMAD1* | Gene |
| rs7670486 | | 4 | 146673371 | *SMAD1* | Gene |
| rs3756021 | | 4 | 146680984 | *SMAD1* | Gene |
| rs11724813 | | 4 | 146689388 | *SMAD1* | Gene |
| rs1016792 | | 4 | 146698229 | *SMAD1* | Gene |
| rs9308190 | | 4 | 146703741 | *SMAD1* | Gene |
| rs2036138 | | 4 | 146704312 | *SMAD1* | Gene |
| rs6848760 | | 4 | 146704447 | *SMAD1* | Gene |
| rs10519979 | | 4 | 149854401 |  | AIM |
| rs2606324 | | 4 | 154871897 |  | GWAS |
| rs2251997 | | 4 | 154873517 |  | GWAS |
| rs2251900 | | 4 | 154873595 |  | GWAS |
| rs17299041 | | 4 | 154892577 |  | GWAS |
| rs1352695 | | 4 | 158919874 |  | AIM |
| rs6552320 | | 4 | 180125429 |  | GWAS |
| rs9998128 | | 4 | 180133701 |  | GWAS |
| rs10520440 | | 4 | 181035999 |  | AIM |
| rs4957114 | | 5 | 1009974 |  | AIM |
| rs257748 | | 5 | 15872615 |  | AIM |
| rs1353251 | | 5 | 35892964 |  | AIM |
| rs13188386 | | 5 | 42509312 |  | Literature |
| rs9292118 | | 5 | 55935953 |  | AIM |
| rs2052550 | | 5 | 78308698 |  | Literature |
| rs173686 | | 5 | 82847256 |  | AIM |
| rs153898 | | 5 | 94214378 |  | AIM |
| rs1990745 | | 5 | 103409821 |  | AIM |
| rs1807912 | | 5 | 109245567 |  | AIM |
| rs1366199 | | 5 | 115349647 |  | AIM |
| rs1021516 | | 5 | 116572071 |  | AIM |
| rs7705743 | | 5 | 118063692 |  | GWAS |
| rs1560550 | | 5 | 121217395 |  | AIM |
| rs330679 | | 5 | 123233993 |  | GWAS |
| rs266642 | | 5 | 125572763 |  | GWAS |
| rs31251 | | 5 | 130861845 |  | AIM |
| rs9327744 | | 5 | 135501661 | *SMAD5* | Gene |
| rs746994 | | 5 | 135510912 | *SMAD5* | Gene |
| rs17169884 | | 5 | 135513296 | *SMAD5* | Gene |
| rs4585442 | | 5 | 135536280 | *SMAD5* | Gene |
| rs6886699 | | 5 | 135543637 | *SMAD5* | Gene |
| rs877826 | | 5 | 138646696 |  | AIM |
| rs10515535 | | 5 | 143496335 |  | AIM |
| rs11960314 | | 5 | 169991805 |  | GWAS |
| rs185493 | | 5 | 177923864 |  | AIM |
| rs1477277 | | 5 | 180607628 |  | AIM |
| rs727056 | | 6 | 170044 |  | AIM |
| rs9504361 | | 6 | 522820 | *GWAS* | GWAS |
| rs12198986 | | 6 | 7665058 | *BMP6* | Gene |
| rs3812163 | | 6 | 7670759 | *BMP6* | Gene |
| rs1107495 | | 6 | 7671056 | *BMP6* | Gene |
| rs270417 | | 6 | 7674613 | *BMP6* | Gene |
| rs7753111 | | 6 | 7675943 | *BMP6* | Gene |
| rs6910759 | | 6 | 7678861 | *BMP6* | Gene |
| rs932659 | | 6 | 7680787 | *BMP6* | Gene |
| rs2068361 | | 6 | 7681742 | *BMP6* | Gene |
| rs270406 | | 6 | 7682807 | *BMP6* | Gene |
| rs911749 | | 6 | 7683958 | *BMP6* | Gene |
| rs1535429 | | 6 | 7687173 | *BMP6* | Gene |
| rs9505270 | | 6 | 7688662 | *BMP6* | Gene |
| rs10452669 | | 6 | 7690851 | *BMP6* | Gene |
| rs17763716 | | 6 | 7691863 | *BMP6* | Gene |
| rs13196371 | | 6 | 7693784 | *BMP6* | Gene |
| rs270413 | | 6 | 7694642 | *BMP6* | Gene |
| rs270377 | | 6 | 7708017 | *BMP6* | Gene |
| rs1226102 | | 6 | 7708113 | *BMP6* | Gene |
| rs270398 | | 6 | 7710839 | *BMP6* | Gene |
| rs270392 | | 6 | 7715696 | *BMP6* | Gene |
| rs1885448 | | 6 | 7716777 | *BMP6* | Gene |
| rs270386 | | 6 | 7718968 | *BMP6* | Gene |
| rs270383 | | 6 | 7720680 | *BMP6* | Gene |
| rs189727 | | 6 | 7721016 | *BMP6* | Gene |
| rs11243205 | | 6 | 7725435 | *BMP6* | Gene |
| rs9328444 | | 6 | 7730458 | *BMP6* | Gene |
| rs9379137 | | 6 | 7733601 | *BMP6* | Gene |
| rs12210175 | | 6 | 7741654 | *BMP6* | Gene |
| rs1150890 | | 6 | 7742112 | *BMP6* | Gene |
| rs927406 | 6 | 7752303 | *BMP6* | Gene |  |
| rs267802 | 6 | 7760518 | *BMP6* | Gene |  |
| rs198354 | 6 | 7762432 | *BMP6* | Gene |  |
| rs11964227 | 6 | 7765668 | *BMP6* | Gene |  |
| rs267174 | 6 | 7768578 | *BMP6* | Gene |  |
| rs267175 | 6 | 7768910 | *BMP6* | Gene |  |
| rs267177 | 6 | 7773541 | *BMP6* | Gene |  |
| rs267180 | 6 | 7775140 | *BMP6* | Gene |  |
| rs267183 | 6 | 7777469 | *BMP6* | Gene |  |
| rs267184 | 6 | 7778428 | *BMP6* | Gene |  |
| rs267190 | 6 | 7787120 | *BMP6* | Gene |  |
| rs267191 | 6 | 7787306 | *BMP6* | Gene |  |
| rs169124 | 6 | 7792842 | *BMP6* | Gene |  |
| rs267196 | 6 | 7794334 | *BMP6* | Gene |  |
| rs267202 | 6 | 7799235 | *BMP6* | Gene |  |
| rs267205 | 6 | 7805316 | *BMP6* | Gene |  |
| rs267207 | 6 | 7806194 | *BMP6* | Gene |  |
| rs408505 | 6 | 7811426 | *BMP6* | Gene |  |
| rs1235192 | 6 | 7812045 | *BMP6* | Gene |  |
| rs1237087 | 6 | 7813865 | *BMP6* | Gene |  |
| rs9505293 | 6 | 7815592 | *BMP6* | Gene |  |
| rs752751 | 6 | 7816613 | *BMP6* | Gene |  |
| rs592849 | 6 | 7817468 | *BMP6* | Gene |  |
| rs1225929 | 6 | 7819232 | *BMP6* | Gene |  |
| rs1225933 | 6 | 7821214 | *BMP6* | Gene |  |
| rs1225934 | 6 | 7822418 | *BMP6* | Gene |  |
| rs267170 | 6 | 7823565 | *BMP6* | Gene |  |
| rs1044104 | 6 | 7826310 | *BMP6* | Gene |  |
| rs7764128 | 6 | 7827204 | *BMP6* | Gene |  |
| rs8643 | 6 | 7828072 | *BMP6* | Gene |  |
| rs11962800 | 6 | 7831904 | *BMP6* | Gene |  |
| rs6911727 | 6 | 9061397 |  | AIM |  |
| rs767022 | 6 | 9084652 |  | AIM |  |
| rs1953088 | 6 | 13033922 |  | AIM |  |
| rs10498725 | 6 | 25562994 |  | Literature |  |
| rs2274089 | 6 | 25596562 |  | Literature |  |
| rs932316 | 6 | 25749179 |  | Literature |  |
| rs17270561 | 6 | 25928418 |  | Literature |  |
| rs12216125 | 6 | 26105437 |  | Literature |  |
| rs9379818 | 6 | 26131185 |  | Literature |  |
| rs2794719 | 6 | 26196869 | *HFE* | Gene |  |
| rs9366637 | 6 | 26197077 | *HFE* | Gene |  |
| rs1799945 | 6 | 26199158 |  | Literature |  |
| rs2071303 | 6 | 26199315 | *HFE* | Gene |  |
| rs1800562 | 6 | 26201120 |  | Gene and Literature |  |
| rs1800708 | 6 | 26201282 | *HFE* | Gene |  |
| rs2858996 | 6 | 26202005 | *HFE* | Gene |  |
| rs1572982 | 6 | 26202346 | *HFE* | Gene |  |
| rs707889 | 6 | 26203910 | *HFE* | Gene |  |
| rs17596719 | 6 | 26205173 | *HFE* | Gene |  |
| rs13194984 | 6 | 26608542 |  | Literature |  |
| rs13194491 | 6 | 27145059 |  | Literature |  |
| rs2857708 | 6 | 31641585 | *TNF* | Gene |  |
| rs2844484 | 6 | 31644203 | *TNF* | Gene |  |
| rs2844482 | 6 | 31647746 | *TNF* | Gene |  |
| rs2229094 | 6 | 31648535 | *TNF* | Gene |  |
| rs1799964 | 6 | 31650287 | *TNF* | Gene |  |
| rs1800629 | 6 | 31651010 |  | Literature |  |
| rs3093661 | 6 | 31651737 | *TNF* | Gene |  |
| rs3093662 | 6 | 31652168 | *TNF* | Gene |  |
| rs3093553 | 6 | 31657535 | *TNF* | Gene |  |
| rs7772436 | 6 | 34099346 |  | GWAS |  |
| rs10484578 | 6 | 35354297 |  | AIM |  |
| rs12665229 | 6 | 38235569 | *BTBD9* | Gene |  |
| rs2073021 | 6 | 38239420 | *BTBD9* | Gene |  |
| rs12663450 | 6 | 38243708 | *BTBD9* | Gene |  |
| rs13198420 | 6 | 38247460 | *BTBD9* | Gene |  |
| rs12206712 | 6 | 38247726 | *BTBD9* | Gene |  |
| rs737172 | 6 | 38248609 | *BTBD9* | Gene |  |
| rs3800358 | 6 | 38249982 | *BTBD9* | Gene |  |
| rs11754110 | 6 | 38252296 | *BTBD9* | Gene |  |
| rs10947714 | 6 | 38252702 | *BTBD9* | Gene |  |
| rs17678747 | 6 | 38253943 | *BTBD9* | Gene |  |
| rs7742915 | 6 | 38255723 | *BTBD9* | Gene |  |
| rs9470813 | 6 | 38259833 | *BTBD9* | Gene |  |
| rs11757846 | 6 | 38262919 | *BTBD9* | Gene |  |
| rs10807192 | 6 | 38267796 | *BTBD9* | Gene |  |
| rs9470825 | 6 | 38270288 | *BTBD9* | Gene |  |
| rs10456461 | 6 | 38274417 | *BTBD9* | Gene |  |
| rs10456462 | 6 | 38274489 | *BTBD9* | Gene |  |
| rs1885323 | 6 | 38275272 | *BTBD9* | Gene |  |
| rs1931765 | 6 | 38282799 | *BTBD9* | Gene |  |
| rs12214213 | 6 | 38283650 | *BTBD9* | Gene |  |
| rs16890436 | 6 | 38284692 | *BTBD9* | Gene |  |
| rs4299828 | 6 | 38285645 | *BTBD9* | Gene |  |
| rs4714131 | 6 | 38297014 | *BTBD9* | Gene |  |
| rs12526152 | 6 | 38307466 | *BTBD9* | Gene |  |
| rs9296239 | 6 | 38315614 | *BTBD9* | Gene |  |
| rs9296240 | 6 | 38318497 | *BTBD9* | Gene |  |
| rs1118268 | 6 | 38321069 | *BTBD9* | Gene |  |
| rs4714135 | 6 | 38324372 | *BTBD9* | Gene |  |
| rs9366950 | 6 | 38324672 | *BTBD9* | Gene |  |
| rs726160 | 6 | 38329617 | *BTBD9* | Gene |  |
| rs10947723 | 6 | 38329996 | *BTBD9* | Gene |  |
| rs12191476 | 6 | 38331364 | *BTBD9* | Gene |  |
| rs17685625 | 6 | 38345503 | *BTBD9* | Gene |  |
| rs9470848 | 6 | 38348063 | *BTBD9* | Gene |  |
| rs9462426 | 6 | 38354751 | *BTBD9* | Gene |  |
| rs7739762 | 6 | 38355698 | *BTBD9* | Gene |  |
| rs9470850 | 6 | 38356173 | *BTBD9* | Gene |  |
| rs4711531 | 6 | 38358758 | *BTBD9* | Gene |  |
| rs12206324 | 6 | 38365813 | *BTBD9* | Gene |  |
| rs12208912 | 6 | 38380861 | *BTBD9* | Gene |  |
| rs4714144 | 6 | 38381069 | *BTBD9* | Gene |  |
| rs6905637 | 6 | 38385888 | *BTBD9* | Gene |  |
| rs872234 | 6 | 38397782 | *BTBD9* | Gene |  |
| rs228184 | 6 | 38400595 | *BTBD9* | Gene |  |
| rs228181 | 6 | 38408118 | *BTBD9* | Gene |  |
| rs6938840 | 6 | 38412833 | *BTBD9* | Gene |  |
| rs228185 | 6 | 38423194 | *BTBD9* | Gene |  |
| rs228188 | 6 | 38426351 | *BTBD9* | Gene |  |
| rs2179533 | 6 | 38426699 | *BTBD9* | Gene |  |
| rs12055513 | 6 | 38429507 | *BTBD9* | Gene |  |
| rs4714148 | 6 | 38431277 | *BTBD9* | Gene |  |
| rs4573069 | 6 | 38431723 | *BTBD9* | Gene |  |
| rs9394492 | 6 | 38440588 | *BTBD9* | Gene |  |
| rs1883610 | 6 | 38448077 | *BTBD9* | Gene |  |
| rs4711542 | 6 | 38450140 | *BTBD9* | Gene |  |
| rs4714152 | 6 | 38451319 | *BTBD9* | Gene |  |
| rs4714156 | 6 | 38469090 | *BTBD9* | Gene |  |
| rs9296249 | 6 | 38473819 | *BTBD9* | Gene |  |
| rs4711546 | 6 | 38474164 | *BTBD9* | Gene |  |
| rs10484927 | 6 | 38481607 | *BTBD9* | Gene |  |
| rs2814896 | 6 | 38488782 | *BTBD9* | Gene |  |
| rs2745379 | 6 | 38489086 | *BTBD9* | Gene |  |
| rs2814888 | 6 | 38489334 | *BTBD9* | Gene |  |
| rs4623233 | 6 | 38522365 | *BTBD9* | Gene |  |
| rs3923809 | 6 | 38548948 | *BTBD9* | Gene |  |
| rs4236058 | 6 | 38550426 | *BTBD9* | Gene |  |
| rs9470885 | 6 | 38553377 | *BTBD9* | Gene |  |
| rs12200371 | 6 | 38554612 | *BTBD9* | Gene |  |
| rs12206905 | 6 | 38575642 | *BTBD9* | Gene |  |
| rs4316001 | 6 | 38576865 | *BTBD9* | Gene |  |
| rs10947739 | 6 | 38577301 | *BTBD9* | Gene |  |
| rs4236060 | 6 | 38578065 | *BTBD9* | Gene |  |
| rs4711549 | 6 | 38582641 | *BTBD9* | Gene |  |
| rs6923737 | 6 | 38591542 | *BTBD9* | Gene |  |
| rs9394508 | 6 | 38594615 | *BTBD9* | Gene |  |
| rs6924443 | 6 | 38594856 | *BTBD9* | Gene |  |
| rs12525647 | 6 | 38595004 | *BTBD9* | Gene |  |
| rs4141854 | 6 | 38615490 | *BTBD9* | Gene |  |
| rs6931131 | 6 | 38623973 | *BTBD9* | Gene |  |
| rs4254983 | 6 | 38625544 | *BTBD9* | Gene |  |
| rs2814889 | 6 | 38627739 | *BTBD9* | Gene |  |
| rs9296252 | 6 | 38629422 | *BTBD9* | Gene |  |
| rs17614684 | 6 | 38630608 | *BTBD9* | Gene |  |
| rs2814890 | 6 | 38638808 | *BTBD9* | Gene |  |
| rs2814891 | 6 | 38644949 | *BTBD9* | Gene |  |
| rs2814894 | 6 | 38652475 | *BTBD9* | Gene |  |
| rs2748166 | 6 | 38660252 | *BTBD9* | Gene |  |
| rs12525759 | 6 | 38660482 | *BTBD9* | Gene |  |
| rs2180106 | 6 | 38668193 | *BTBD9* | Gene |  |
| rs910516 | 6 | 38672926 | *BTBD9* | Gene |  |
| rs1699015 | 6 | 38680568 | *BTBD9* | Gene |  |
| rs4714173 | 6 | 38710250 | *BTBD9* | Gene |  |
| rs1781708 | 6 | 38712732 | *BTBD9* | Gene |  |
| rs1781742 | 6 | 38713353 | *BTBD9* | Gene |  |
| rs3734693 | 6 | 44073143 |  | AIM |  |
| rs1928533 | 6 | 45617802 |  | AIM |  |
| rs966707 | 6 | 51736178 |  | AIM |  |
| rs1993118 | 6 | 57005993 |  | GWAS |  |
| rs1016461 | 6 | 69092970 |  | AIM |  |
| rs1338248 | 6 | 93639259 |  | AIM |  |
| rs1268055 | 6 | 109289637 |  | GWAS |  |
| rs1260658 | 6 | 109526918 |  | AIM |  |
| rs1541317 | 6 | 118130009 |  | AIM |  |
| rs9320808 | 6 | 121696295 |  | AIM |  |
| rs7760517 | 6 | 123809909 |  | GWAS |  |
| rs1538956 | 6 | 127005719 |  | AIM |  |
| rs1022573 | 6 | 127096607 |  | AIM |  |
| rs6569792 | 6 | 132736444 |  | AIM |  |
| rs7745902 | 6 | 132878166 |  | GWAS |  |
| rs839556 | 6 | 142913571 |  | AIM |  |
| rs200148 | 6 | 143387389 |  | AIM |  |
| rs6940739 | 6 | 157335479 |  | GWAS |  |
| rs2356043 | 6 | 157338874 |  | GWAS |  |
| rs9295316 | 6 | 158487054 |  | AIM |  |
| rs4516970 | 6 | 160057677 |  | Literature |  |
| rs727619 | 6 | 170623826 |  | AIM |  |
| rs6979676 | 7 | 6021513 | *EIF2AK1* | Gene |  |
| rs7803611 | 7 | 6022232 | *EIF2AK1* | Gene |  |
| rs3779106 | 7 | 6022408 | *EIF2AK1* | Gene |  |
| rs3779108 | 7 | 6022683 | *EIF2AK1* | Gene |  |
| rs1860459 | 7 | 6023145 | *EIF2AK1* | Gene |  |
| rs3801030 | 7 | 6028755 | *EIF2AK1* | Gene |  |
| rs7781199 | 7 | 6029354 | *EIF2AK1* | Gene |  |
| rs10249759 | 7 | 6032272 | *EIF2AK1* | Gene |  |
| rs10255418 | 7 | 6037382 | *EIF2AK1* | Gene |  |
| rs4724770 | 7 | 6037803 | *EIF2AK1* | Gene |  |
| rs12672450 | 7 | 6040725 | *EIF2AK1* | Gene |  |
| rs17136348 | 7 | 6041235 | *EIF2AK1* | Gene |  |
| rs2302332 | 7 | 6044002 | *EIF2AK1* | Gene |  |
| rs2302330 | 7 | 6044994 | *EIF2AK1* | Gene |  |
| rs10249956 | 7 | 6056357 | *EIF2AK1* | Gene |  |
| rs475323 | 7 | 6057126 | *EIF2AK1* | Gene |  |
| rs852154 | 7 | 6057253 | *EIF2AK1* | Gene |  |
| rs10263415 | 7 | 7691330 |  | GWAS |  |
| rs4725084 | 7 | 8343391 |  | GWAS |  |
| rs6943135 | 7 | 9797770 |  | GWAS |  |
| rs7787204 | 7 | 9813821 |  | GWAS |  |
| rs1709592 | 7 | 9836722 |  | GWAS |  |
| rs6963591 | 7 | 22723795 | *IL6* | Gene |  |
| rs7805828 | 7 | 22725087 | *IL6* | Gene |  |
| rs1880242 | 7 | 22726132 | *IL6* | Gene |  |
| rs2056576 | 7 | 22727727 | *IL6* | Gene |  |
| rs12700386 | 7 | 22729534 | *IL6* | Gene |  |
| rs1474347 | 7 | 22734649 | *IL6* | Gene |  |
| rs2066992 | 7 | 22734774 | *IL6* | Gene |  |
| rs2069840 | 7 | 22735097 | *IL6* | Gene |  |
| rs1554606 | 7 | 22735232 | *IL6* | Gene |  |
| rs2069852 | 7 | 22738785 | *IL6* | Gene |  |
| rs10242595 | 7 | 22740756 | *IL6* | Gene |  |
| rs10486576 | 7 | 28085668 |  | AIM |  |
| rs740250 | 7 | 28963469 |  | GWAS |  |
| rs29880 | 7 | 40921006 |  | Literature |  |
| rs10248051 | 7 | 51086847 |  | AIM |  |
| rs10214949 | 7 | 78886529 |  | AIM |  |
| rs7805730 | 7 | 84826619 |  | GWAS |  |
| rs7790941 | 7 | 84838794 |  | GWAS |  |
| rs722263 | 7 | 92531463 |  | AIM |  |
| rs3294 | 7 | 98293016 |  | AIM |  |
| rs7786877 | 7 | 100051951 | *TFR2* | Gene |  |
| rs10487157 | 7 | 100052423 | *TFR2* | Gene |  |
| rs7457868 | 7 | 100065124 | *TFR2* | Gene |  |
| rs4434553 | 7 | 100078127 | *TFR2* | Gene |  |
| rs2075672 | 7 | 100078232 | *TFR2* | Gene |  |
| rs201492 | 7 | 101347573 |  | AIM |  |
| rs4727586 | 7 | 103494172 |  | GWAS |  |
| rs10488172 | 7 | 132985716 |  | AIM |  |
| rs802524 | 7 | 145582575 |  | AIM |  |
| rs963314 | 7 | 147300280 |  | AIM |  |
| rs2442567 | 8 | 6445077 |  | AIM |  |
| rs981905 | 8 | 14126601 |  | GWAS |  |
| rs10087468 | 8 | 14128982 |  | GWAS tag SNP |  |
| rs6989764 | 8 | 14129590 |  | GWAS tag SNP |  |
| rs34586769 | 8 | 14129768 |  | GWAS tag SNP |  |
| rs4637829 | 8 | 14132498 |  | GWAS |  |
| rs13255607 | 8 | 14132722 |  | GWAS tag SNP |  |
| rs4831559 | 8 | 14134101 |  | GWAS tag SNP |  |
| rs17118836 | 8 | 14136145 |  | GWAS tag SNP |  |
| rs13266960 | 8 | 14137038 |  | GWAS tag SNP |  |
| rs1382142 | 8 | 14138300 |  | GWAS tag SNP |  |
| rs6530737 | 8 | 14140134 |  | GWAS tag SNP |  |
| rs17118860 | 8 | 14142476 |  | GWAS tag SNP |  |
| rs9325872 | 8 | 20524551 |  | AIM |  |
| rs11136002 | 8 | 22273027 | *SLC39A14* | Gene |  |
| rs13258195 | 8 | 22283216 | *SLC39A14* | Gene |  |
| rs4872479 | 8 | 22289581 | *SLC39A14* | Gene |  |
| rs12545568 | 8 | 22295813 | *SLC39A14* | Gene |  |
| rs11783730 | 8 | 22300842 | *SLC39A14* | Gene |  |
| rs7844699 | 8 | 22304865 | *SLC39A14* | Gene |  |
| rs1568569 | 8 | 22305888 | *SLC39A14* | Gene |  |
| rs7818776 | 8 | 22308255 | *SLC39A14* | Gene |  |
| rs11136029 | 8 | 22309430 | *SLC39A14* | Gene |  |
| rs7833302 | 8 | 22312255 | *SLC39A14* | Gene |  |
| rs4871996 | 8 | 22313247 | *SLC39A14* | Gene |  |
| rs750668 | 8 | 22313489 | *SLC39A14* | Gene |  |
| rs870215 | 8 | 22314082 | *SLC39A14* | Gene |  |
| rs11987482 | 8 | 22317574 | *SLC39A14* | Gene |  |
| rs2293144 | 8 | 22318363 | *SLC39A14* | Gene |  |
| rs17060826 | 8 | 22319117 | *SLC39A14* | Gene |  |
| rs13259873 | 8 | 22322237 | *SLC39A14* | Gene |  |
| rs11989798 | 8 | 22326597 | *SLC39A14* | Gene |  |
| rs2280521 | 8 | 22327940 | *SLC39A14* | Gene |  |
| rs2280522 | 8 | 22328171 | *SLC39A14* | Gene |  |
| rs6558052 | 8 | 22329632 | *SLC39A14* | Gene |  |
| rs12545575 | 8 | 22330562 | *SLC39A14* | Gene |  |
| rs10101909 | 8 | 22332985 | *SLC39A14* | Gene |  |
| rs17060854 | 8 | 22333856 | *SLC39A14* | Gene |  |
| rs1051638 | 8 | 22334726 | *SLC39A14* | Gene |  |
| rs1051708 | 8 | 22335808 | *SLC39A14* | Gene |  |
| rs3943520 | 8 | 22338677 | *SLC39A14* | Gene |  |
| rs972575 | 8 | 23438539 | *SLC25A37* | Gene |  |
| rs6557678 | 8 | 23438930 | *SLC25A37* | Gene |  |
| rs6557679 | 8 | 23439688 | *SLC25A37* | Gene |  |
| rs721183 | 8 | 23441779 | *SLC25A37* | Gene |  |
| rs2002508 | 8 | 23445264 | *SLC25A37* | Gene |  |
| rs11781222 | 8 | 23445516 | *SLC25A37* | Gene |  |
| rs1008856 | 8 | 23450853 | *SLC25A37* | Gene |  |
| rs10503725 | 8 | 23452482 | *SLC25A37* | Gene |  |
| rs4593549 | 8 | 23452677 | *SLC25A37* | Gene |  |
| rs10503726 | 8 | 23453211 | *SLC25A37* | Gene |  |
| rs4871881 | 8 | 23453709 | *SLC25A37* | Gene |  |
| rs2872716 | 8 | 23454378 | *SLC25A37* | Gene |  |
| rs7015818 | 8 | 23458015 | *SLC25A37* | Gene |  |
| rs2280861 | 8 | 23460730 | *SLC25A37* | Gene |  |
| rs2928672 | 8 | 23466270 | *SLC25A37* | Gene |  |
| rs17089358 | 8 | 23467220 | *SLC25A37* | Gene |  |
| rs7816824 | 8 | 23467743 | *SLC25A37* | Gene |  |
| rs4872154 | 8 | 23469280 | *SLC25A37* | Gene |  |
| rs13254494 | 8 | 23470688 | *SLC25A37* | Gene |  |
| rs2137304 | 8 | 23471185 | *SLC25A37* | Gene |  |
| rs11778179 | 8 | 23471600 | *SLC25A37* | Gene |  |
| rs2978487 | 8 | 23471979 | *SLC25A37* | Gene |  |
| rs2942202 | 8 | 23474389 | *SLC25A37* | Gene |  |
| rs7830129 | 8 | 23475814 | *SLC25A37* | Gene |  |
| rs2978475 | 8 | 23476571 | *SLC25A37* | Gene |  |
| rs2928686 | 8 | 23477641 | *SLC25A37* | Gene |  |
| rs2978477 | 8 | 23479318 | *SLC25A37* | Gene |  |
| rs2942194 | 8 | 23479614 | *SLC25A37* | Gene |  |
| rs2942213 | 8 | 23483864 | *SLC25A37* | Gene |  |
| rs1047384 | 8 | 23485274 | *SLC25A37* | Gene |  |
| rs10104250 | 8 | 23487137 | *SLC25A37* | Gene |  |
| rs3174040 | 8 | 23487352 | *SLC25A37* | Gene |  |
| rs8534 | 8 | 23488277 | *SLC25A37* | Gene |  |
| rs10092233 | 8 | 23490677 | *SLC25A37* | Gene |  |
| rs1457271 | 8 | 24778989 |  | GWAS |  |
| rs7844382 | 8 | 24785810 |  | GWAS |  |
| rs7463344 | 8 | 33983069 |  | AIM |  |
| rs718251 | 8 | 52877076 |  | AIM |  |
| rs884839 | 8 | 67182542 |  | AIM |  |
| rs1440369 | 8 | 73728570 |  | AIM |  |
| rs4130405 | 8 | 99489951 |  | AIM |  |
| rs2460970 | 8 | 119698635 |  | GWAS |  |
| rs880034 | 8 | 119702442 |  | GWAS |  |
| rs769322 | 8 | 119738549 |  | AIM |  |
| rs2124036 | 8 | 126717316 |  | AIM |  |
| rs4733652 | 8 | 129913709 |  | AIM |  |
| rs4246828 | 8 | 144240466 |  | AIM |  |
| rs3750203 | 8 | 144803169 |  | AIM |  |
| rs913258 | 9 | 4867246 |  | AIM |  |
| rs7864782 | 9 | 4966029 | *JAK2* | Gene |  |
| rs1887427 | 9 | 4969730 | *JAK2* | Gene |  |
| rs10758669 | 9 | 4971602 | *JAK2* | Gene |  |
| rs1887429 | 9 | 4974549 | *JAK2* | Gene |  |
| rs2274471 | 9 | 4975879 | *JAK2* | Gene |  |
| rs7849191 | 9 | 4978761 | *JAK2* | Gene |  |
| rs7030260 | 9 | 4998070 | *JAK2* | Gene |  |
| rs10121491 | 9 | 5036935 | *JAK2* | Gene |  |
| rs1328917 | 9 | 5039065 | *JAK2* | Gene |  |
| rs2149555 | 9 | 5043743 | *JAK2* | Gene |  |
| rs1536800 | 9 | 5045434 | *JAK2* | Gene |  |
| rs2149556 | 9 | 5049440 | *JAK2* | Gene |  |
| rs7859390 | 9 | 5052473 | *JAK2* | Gene |  |
| rs913594 | 9 | 5053199 | *JAK2* | Gene |  |
| rs12339666 | 9 | 5053296 | *JAK2* | Gene |  |
| rs3780367 | 9 | 5058755 | *JAK2* | Gene |  |
| rs10974947 | 9 | 5062846 | *JAK2* | Gene |  |
| rs2031904 | 9 | 5077087 | *JAK2* | Gene |  |
| rs3824432 | 9 | 5081675 | *JAK2* | Gene |  |
| rs7847294 | 9 | 5087281 | *JAK2* | Gene |  |
| rs10815157 | 9 | 5098771 | *JAK2* | Gene |  |
| rs17425819 | 9 | 5104773 | *JAK2* | Gene |  |
| rs10815160 | 9 | 5106616 | *JAK2* | Gene |  |
| rs2151065 | 9 | 16235716 |  | AIM |  |
| rs2840290 | 9 | 16723957 |  | AIM |  |
| rs1487499 | 9 | 17004026 |  | GWAS |  |
| rs1231335 | 9 | 25715318 |  | GWAS |  |
| rs10812768 | 9 | 28253705 |  | GWAS |  |
| rs10968467 | 9 | 28288064 |  | GWAS |  |
| rs10970947 | 9 | 32365432 | *ACO1* | Gene |  |
| rs1023087 | 9 | 32366618 | *ACO1* | Gene |  |
| rs867469 | 9 | 32373708 | *ACO1* | Gene |  |
| rs1028932 | 9 | 32381784 | *ACO1* | Gene |  |
| rs1041321 | 9 | 32384422 | *ACO1* | Gene |  |
| rs13302577 | 9 | 32385896 | *ACO1* | Gene |  |
| rs10813808 | 9 | 32387920 | *ACO1* | Gene |  |
| rs6476361 | 9 | 32391610 | *ACO1* | Gene |  |
| rs4879584 | 9 | 32402622 | *ACO1* | Gene |  |
| rs10435797 | 9 | 32407621 | *ACO1* | Gene |  |
| rs2026739 | 9 | 32408237 | *ACO1* | Gene |  |
| rs7026133 | 9 | 32409461 | *ACO1* | Gene |  |
| rs7032871 | 9 | 32412690 | *ACO1* | Gene |  |
| rs10970972 | 9 | 32414210 | *ACO1* | Gene |  |
| rs10813814 | 9 | 32414893 | *ACO1* | Gene |  |
| rs13293491 | 9 | 32415270 | *ACO1* | Gene |  |
| rs3780474 | 9 | 32415676 | *ACO1* | Gene |  |
| rs3780473 | 9 | 32415910 | *ACO1* | Gene and Literature |  |
| rs1556138 | 9 | 32422565 | *ACO1* | Gene |  |
| rs1467713 | 9 | 32422647 | *ACO1* | Gene |  |
| rs10758139 | 9 | 32425676 | *ACO1* | Gene |  |
| rs7022554 | 9 | 32426014 | *ACO1* | Gene |  |
| rs10813816 | 9 | 32427249 | *ACO1* | Gene |  |
| rs10970978 | 9 | 32432256 | *ACO1* | Gene |  |
| rs10813818 | 9 | 32439244 | *ACO1* | Gene |  |
| rs12985 | 9 | 32440187 | *ACO1* | Gene |  |
| rs7042042 | 9 | 32441144 | *ACO1* | Gene |  |
| rs17288914 | 9 | 32442627 | *ACO1* | Gene |  |
| rs10970986 | 9 | 32443278 | *ACO1* | Gene |  |
| rs10869745 | 9 | 70832319 | *FXN* | Gene |  |
| rs3763608 | 9 | 70837195 | *FXN* | Gene |  |
| rs7046232 | 9 | 70837903 | *FXN* | Gene |  |
| rs12000125 | 9 | 70839284 | *FXN* | Gene |  |
| rs1800651 | 9 | 70841425 | *FXN* | Gene |  |
| rs7871596 | 9 | 70847217 | *FXN* | Gene |  |
| rs9411170 | 9 | 70848912 | *FXN* | Gene |  |
| rs3793451 | 9 | 70849100 | *FXN* | Gene |  |
| rs3829062 | 9 | 70858017 | *FXN* | Gene |  |
| rs2309393 | 9 | 70865908 | *FXN* | Gene |  |
| rs3793465 | 9 | 70869258 | *FXN* | Gene |  |
| rs2498431 | 9 | 70874411 | *FXN* | Gene |  |
| rs2498430 | 9 | 70874522 | *FXN* | Gene |  |
| rs9314854 | 9 | 70876001 | *FXN* | Gene |  |
| rs7870295 | 9 | 70876296 | *FXN* | Gene |  |
| rs10890 | 9 | 70877744 | *FXN* | Gene |  |
| rs11145043 | 9 | 70878296 | *FXN* | Gene |  |
| rs17060788 | 9 | 70878597 | *FXN* | Gene |  |
| rs4013967 | 9 | 76086890 |  | AIM |  |
| rs11145300 | 9 | 78956536 |  | GWAS tag SNP |  |
| rs2377887 | 9 | 78960218 |  | GWAS tag SNP |  |
| rs4618761 | 9 | 78960330 |  | GWAS tag SNP |  |
| rs12338171 | 9 | 78962435 |  | GWAS tag SNP |  |
| rs7873307 | 9 | 78963600 |  | GWAS tag SNP |  |
| rs10747014 | 9 | 78965275 |  | GWAS tag SNP |  |
| rs10512064 | 9 | 78966152 |  | GWAS |  |
| rs7022271 | 9 | 78966631 |  | GWAS tag SNP |  |
| rs7869194 | 9 | 78968067 |  | GWAS tag SNP |  |
| rs12156507 | 9 | 78973604 |  | GWAS tag SNP |  |
| rs17723837 | 9 | 78976295 |  | GWAS tag SNP |  |
| rs12342840 | 9 | 78977047 |  | GWAS tag SNP |  |
| rs1536950 | 9 | 99373593 |  | GWAS |  |
| rs2026999 | 9 | 100219712 |  | AIM |  |
| rs10491654 | 9 | 101179348 |  | AIM |  |
| rs1571142 | 9 | 129700631 |  | Literature |  |
| rs6478823 | 9 | 129946669 | *LCN2* | Gene |  |
| rs10987895 | 9 | 129949150 | *LCN2* | Gene |  |
| rs3814526 | 9 | 129950508 | *LCN2* | Gene |  |
| rs10987899 | 9 | 129958191 | *LCN2* | Gene |  |
| rs1215972 | 9 | 132340429 |  | GWAS |  |
| rs7860423 | 9 | 138231384 |  | AIM |  |
| rs10508349 | 10 | 8338970 |  | AIM |  |
| rs7904368 | 10 | 16898593 | *CUBN* | Gene |  |
| rs2603794 | 10 | 16900069 | *CUBN* | Gene |  |
| rs3808925 | 10 | 16900245 | *CUBN* | Gene |  |
| rs780632 | 10 | 16901406 | *CUBN* | Gene |  |
| rs6602161 | 10 | 16902286 | *CUBN* | Gene |  |
| rs2603796 | 10 | 16911462 | *CUBN* | Gene |  |
| rs7087360 | 10 | 16912343 | *CUBN* | Gene |  |
| rs3740169 | 10 | 16913553 | *CUBN* | Gene |  |
| rs2603804 | 10 | 16913750 | *CUBN* | Gene |  |
| rs1797081 | 10 | 16914571 | *CUBN* | Gene |  |
| rs10904824 | 10 | 16914755 | *CUBN* | Gene |  |
| rs4088454 | 10 | 16915198 | *CUBN* | Gene |  |
| rs7897625 | 10 | 16916113 | *CUBN* | Gene |  |
| rs703062 | 10 | 16918210 | *CUBN* | Gene |  |
| rs780831 | 10 | 16924379 | *CUBN* | Gene |  |
| rs780829 | 10 | 16925664 | *CUBN* | Gene |  |
| rs780827 | 10 | 16926812 | *CUBN* | Gene |  |
| rs11254244 | 10 | 16927928 | *CUBN* | Gene |  |
| rs12251746 | 10 | 16931474 | *CUBN* | Gene |  |
| rs7893395 | 10 | 16945506 | *CUBN* | Gene |  |
| rs10752062 | 10 | 16946514 | *CUBN* | Gene |  |
| rs17139378 | 10 | 16949595 | *CUBN* | Gene |  |
| rs703075 | 10 | 16951611 | *CUBN* | Gene |  |
| rs780825 | 10 | 16952140 | *CUBN* | Gene |  |
| rs17139411 | 10 | 16957987 | *CUBN* | Gene |  |
| rs1801241 | 10 | 16958978 | *CUBN* | Gene |  |
| rs780838 | 10 | 16961376 | *CUBN* | Gene |  |
| rs780837 | 10 | 16963396 | *CUBN* | Gene |  |
| rs2796832 | 10 | 16965886 | *CUBN* | Gene |  |
| rs780849 | 10 | 16966739 | *CUBN* | Gene |  |
| rs812975 | 10 | 16966928 | *CUBN* | Gene |  |
| rs809698 | 10 | 16967033 | *CUBN* | Gene |  |
| rs780844 | 10 | 16971471 | *CUBN* | Gene |  |
| rs2669149 | 10 | 16974176 | *CUBN* | Gene |  |
| rs11254267 | 10 | 16976906 | *CUBN* | Gene |  |
| rs780816 | 10 | 16978244 | *CUBN* | Gene |  |
| rs780811 | 10 | 16979554 | *CUBN* | Gene |  |
| rs780810 | 10 | 16979901 | *CUBN* | Gene |  |
| rs10904833 | 10 | 16979982 | *CUBN* | Gene |  |
| rs780807 | 10 | 16988183 | *CUBN* | Gene |  |
| rs780855 | 10 | 16994194 | *CUBN* | Gene |  |
| rs11254277 | 10 | 17004449 | *CUBN* | Gene |  |
| rs1810205 | 10 | 17005233 | *CUBN* | Gene |  |
| rs1276717 | 10 | 17009299 | *CUBN* | Gene |  |
| rs1276720 | 10 | 17011432 | *CUBN* | Gene |  |
| rs10904838 | 10 | 17012017 | *CUBN* | Gene |  |
| rs2271461 | 10 | 17015084 | *CUBN* | Gene |  |
| rs11591673 | 10 | 17016232 | *CUBN* | Gene |  |
| rs2883972 | 10 | 17016624 | *CUBN* | Gene |  |
| rs11254284 | 10 | 17018435 | *CUBN* | Gene |  |
| rs2356589 | 10 | 17019572 | *CUBN* | Gene |  |
| rs1801234 | 10 | 17019667 | *CUBN* | Gene |  |
| rs2271463 | 10 | 17022250 | *CUBN* | Gene |  |
| rs7897704 | 10 | 17025165 | *CUBN* | Gene |  |
| rs7912716 | 10 | 17025323 | *CUBN* | Gene |  |
| rs34683673 | 10 | 17027135 | *CUBN* | Gene |  |
| rs2271466 | 10 | 17029433 | *CUBN* | Gene |  |
| rs11814420 | 10 | 17030914 | *CUBN* | Gene |  |
| rs7893507 | 10 | 17033478 | *CUBN* | Gene |  |
| rs11254294 | 10 | 17033583 | *CUBN* | Gene |  |
| rs3847364 | 10 | 17033827 | *CUBN* | Gene |  |
| rs10904850 | 10 | 17037713 | *CUBN* | Gene |  |
| rs11254299 | 10 | 17038509 | *CUBN* | Gene |  |
| rs4335444 | 10 | 17040623 | *CUBN* | Gene |  |
| rs7906242 | 10 | 17041818 | *CUBN* | Gene |  |
| rs1687713 | 10 | 17044715 | *CUBN* | Gene |  |
| rs1707291 | 10 | 17045996 | *CUBN* | Gene |  |
| rs7071422 | 10 | 17046670 | *CUBN* | Gene |  |
| rs2942359 | 10 | 17048428 | *CUBN* | Gene |  |
| rs1707273 | 10 | 17048907 | *CUBN* | Gene |  |
| rs1707281 | 10 | 17050971 | *CUBN* | Gene |  |
| rs1398431 | 10 | 17051181 | *CUBN* | Gene |  |
| rs9633766 | 10 | 17052104 | *CUBN* | Gene |  |
| rs41526049 | 10 | 17052237 | *CUBN* | Gene |  |
| rs1687694 | 10 | 17053198 | *CUBN* | Gene |  |
| rs7079269 | 10 | 17053301 | *CUBN* | Gene |  |
| rs11254305 | 10 | 17054990 | *CUBN* | Gene |  |
| rs11254308 | 10 | 17056343 | *CUBN* | Gene |  |
| rs11594134 | 10 | 17057343 | *CUBN* | Gene |  |
| rs11254314 | 10 | 17058324 | *CUBN* | Gene |  |
| rs3012501 | 10 | 17058684 | *CUBN* | Gene |  |
| rs3012499 | 10 | 17059019 | *CUBN* | Gene |  |
| rs5015063 | 10 | 17062975 | *CUBN* | Gene |  |
| rs1801231 | 10 | 17064509 | *CUBN* | Gene |  |
| rs2291521 | 10 | 17072287 | *CUBN* | Gene |  |
| rs3012477 | 10 | 17073435 | *CUBN* | Gene |  |
| rs3012478 | 10 | 17075400 | *CUBN* | Gene |  |
| rs3012479 | 10 | 17075828 | *CUBN* | Gene |  |
| rs1276711 | 10 | 17078707 | *CUBN* | Gene |  |
| rs11254325 | 10 | 17079815 | *CUBN* | Gene |  |
| rs7900486 | 10 | 17080855 | *CUBN* | Gene |  |
| rs12414709 | 10 | 17081089 | *CUBN* | Gene |  |
| rs7922356 | 10 | 17085717 | *CUBN* | Gene |  |
| rs4748341 | 10 | 17087787 | *CUBN* | Gene |  |
| rs7921244 | 10 | 17090969 | *CUBN* | Gene |  |
| rs11592014 | 10 | 17092961 | *CUBN* | Gene |  |
| rs3740164 | 10 | 17101687 | *CUBN* | Gene |  |
| rs4082518 | 10 | 17103032 | *CUBN* | Gene |  |
| rs3901939 | 10 | 17104465 | *CUBN* | Gene |  |
| rs7897550 | 10 | 17104998 | *CUBN* | Gene |  |
| rs17431426 | 10 | 17108118 | *CUBN* | Gene |  |
| rs4525114 | 10 | 17108281 | *CUBN* | Gene |  |
| rs2356215 | 10 | 17113906 | *CUBN* | Gene |  |
| rs11254336 | 10 | 17115579 | *CUBN* | Gene |  |
| rs17139669 | 10 | 17117461 | *CUBN* | Gene |  |
| rs11254338 | 10 | 17119757 | *CUBN* | Gene |  |
| rs10904865 | 10 | 17120639 | *CUBN* | Gene |  |
| rs7893634 | 10 | 17121145 | *CUBN* | Gene |  |
| rs7082270 | 10 | 17129948 | *CUBN* | Gene |  |
| rs2087631 | 10 | 17135305 | *CUBN* | Gene |  |
| rs9665553 | 10 | 17144179 | *CUBN* | Gene |  |
| rs1907360 | 10 | 17145674 | *CUBN* | Gene |  |
| rs2172081 | 10 | 17152412 | *CUBN* | Gene |  |
| rs12254816 | 10 | 17154794 | *CUBN* | Gene |  |
| rs7075040 | 10 | 17161703 | *CUBN* | Gene |  |
| rs4748346 | 10 | 17163469 | *CUBN* | Gene |  |
| rs10508520 | 10 | 17166884 | *CUBN* | Gene |  |
| rs2356823 | 10 | 17167246 | *CUBN* | Gene |  |
| rs17139747 | 10 | 17167367 | *CUBN* | Gene |  |
| rs11254363 | 10 | 17170699 | *CUBN* | Gene |  |
| rs2145939 | 10 | 17176175 | *CUBN* | Gene |  |
| rs1033765 | 10 | 17181958 | *CUBN* | Gene |  |
| rs12261966 | 10 | 17183006 | *CUBN* | Gene |  |
| rs10795445 | 10 | 17184002 | *CUBN* | Gene |  |
| rs7906195 | 10 | 17185671 | *CUBN* | Gene |  |
| rs10752067 | 10 | 17188898 | *CUBN* | Gene |  |
| rs1801222 | 10 | 17196157 | *CUBN* | Gene |  |
| rs2281649 | 10 | 17196533 | *CUBN* | Gene |  |
| rs7899751 | 10 | 17198296 | *CUBN* | Gene |  |
| rs7900190 | 10 | 17198644 | *CUBN* | Gene |  |
| rs2277210 | 10 | 17202509 | *CUBN* | Gene |  |
| rs17139880 | 10 | 17203878 | *CUBN* | Gene |  |
| rs1914172 | 10 | 17204227 | *CUBN* | Gene |  |
| rs7070148 | 10 | 17204631 | *CUBN* | Gene |  |
| rs2273737 | 10 | 17205558 | *CUBN* | Gene |  |
| rs6602177 | 10 | 17207147 | *CUBN* | Gene |  |
| rs7916688 | 10 | 17207428 | *CUBN* | Gene |  |
| rs932640 | 10 | 17207738 | *CUBN* | Gene |  |
| rs10904881 | 10 | 17212958 | *CUBN* | Gene |  |
| rs359280 | 10 | 17359713 |  | AIM |  |
| rs11813505 | 10 | 24661886 |  | AIM |  |
| rs2297330 | 10 | 24850669 |  | Literature |  |
| rs727345 | 10 | 31939079 |  | AIM |  |
| rs2785279 | 10 | 33749882 |  | AIM |  |
| rs9325886 | 10 | 48032343 | *GDF2* | Gene |  |
| rs7923671 | 10 | 48034576 | *GDF2* | Gene |  |
| rs12252199 | 10 | 48036280 | *GDF2* | Gene |  |
| rs11204215 | 10 | 48039161 | *GDF2* | Gene |  |
| rs11595346 | 10 | 48040198 | *GDF2* | Gene |  |
| rs1268722 | 10 | 50621216 |  | AIM |  |
| rs4934436 | 10 | 90773300 |  | AIM |  |
| rs2490741 | 10 | 94584545 | *EXOC6* | Gene |  |
| rs3740380 | 10 | 94588239 | *EXOC6* | Gene |  |
| rs11516919 | 10 | 94608905 | *EXOC6* | Gene |  |
| rs12769058 | 10 | 94621902 | *EXOC6* | Gene |  |
| rs7069896 | 10 | 94627797 | *EXOC6* | Gene |  |
| rs9633694 | 10 | 94638938 | *EXOC6* | Gene |  |
| rs7923502 | 10 | 94655411 | *EXOC6* | Gene |  |
| rs10786060 | 10 | 94656561 | *EXOC6* | Gene |  |
| rs6583847 | 10 | 94656963 | *EXOC6* | Gene |  |
| rs1326330 | 10 | 94685535 | *EXOC6* | Gene |  |
| rs12415602 | 10 | 94689603 | *EXOC6* | Gene |  |
| rs2274374 | 10 | 94690289 | *EXOC6* | Gene |  |
| rs10509647 | 10 | 94696613 | *EXOC6* | Gene |  |
| rs7075749 | 10 | 94708248 | *EXOC6* | Gene |  |
| rs7069010 | 10 | 94711885 | *EXOC6* | Gene |  |
| rs7082214 | 10 | 94730507 | *EXOC6* | Gene |  |
| rs1571034 | 10 | 94744240 | *EXOC6* | Gene |  |
| rs1409386 | 10 | 94752320 | *EXOC6* | Gene |  |
| rs7081888 | 10 | 94754640 | *EXOC6* | Gene |  |
| rs3736936 | 10 | 94763647 | *EXOC6* | Gene |  |
| rs17108061 | 10 | 94771776 | *EXOC6* | Gene |  |
| rs9419765 | 10 | 94788151 | *EXOC6* | Gene |  |
| rs10748589 | 10 | 94795203 | *EXOC6* | Gene |  |
| rs2486681 | 10 | 94795645 | *EXOC6* | Gene |  |
| rs8211 | 10 | 94809043 | *EXOC6* | Gene |  |
| rs2181019 | 10 | 108301686 |  | Literature |  |
| rs1050755 | 10 | 112043589 |  | AIM |  |
| rs1397618 | 10 | 120822665 |  | AIM |  |
| rs2292692 | 10 | 127727920 | *ADAM12* | Gene |  |
| rs7085290 | 10 | 127826099 | *ADAM12* | Gene |  |
| rs1674914 | 10 | 127834174 | *ADAM12* | Gene |  |
| rs7899821 | 10 | 127920125 | *ADAM12* | Gene |  |
| rs17684825 | 10 | 127947094 | *ADAM12* | Gene |  |
| rs12788345 | 11 | 6407594 | *HPX* | Gene |  |
| rs10500671 | 11 | 6416602 | *HPX* | Gene |  |
| rs2035675 | 11 | 6419664 | *HPX* | Gene |  |
| rs7113742 | 11 | 6422652 | *HPX* | Gene |  |
| rs2595456 | 11 | 6841339 |  | AIM |  |
| rs722317 | 11 | 15880138 |  | AIM |  |
| rs11024066 | 11 | 16854072 |  | GWAS |  |
| rs1109748 | 11 | 61479221 | *FTH1* | Gene |  |
| rs2668898 | 11 | 61482074 | *FTH1* | Gene |  |
| rs195157 | 11 | 61484367 | *FTH1* | Gene |  |
| rs195156 | 11 | 61485639 | *FTH1* | Gene |  |
| rs2073588 | 11 | 61492987 | *FTH1* | Gene |  |
| rs948360 | 11 | 65863301 |  | AIM |  |
| rs7927381 | 11 | 67103319 | *GSTP1* | Gene |  |
| rs614080 | 11 | 67103863 | *GSTP1* | Gene |  |
| rs6591256 | 11 | 67106475 | *GSTP1* | Gene |  |
| rs8191439 | 11 | 67107873 | *GSTP1* | Gene |  |
| rs1695 | 11 | 67109265 |  | Literature |  |
| rs749174 | 11 | 67109829 | *GSTP1* | Gene |  |
| rs947895 | 11 | 67110982 | *GSTP1* | Gene |  |
| rs12419334 | 11 | 70817120 | *DHCR7* | Gene |  |
| rs1792275 | 11 | 70817804 | *DHCR7* | Gene |  |
| rs1792273 | 11 | 70818134 | *DHCR7* | Gene |  |
| rs1790349 | 11 | 70819998 | *DHCR7* | Gene |  |
| rs12797951 | 11 | 70820914 | *DHCR7* | Gene |  |
| rs12422045 | 11 | 70832468 | *DHCR7* | Gene |  |
| rs4316537 | 11 | 70832777 | *DHCR7* | Gene |  |
| rs3750997 | 11 | 70836489 | *DHCR7* | Gene |  |
| rs2002064 | 11 | 70841068 | *DHCR7* | Gene |  |
| rs1149580 | 11 | 76227161 |  | GWAS |  |
| rs1225138 | 11 | 76231135 |  | GWAS |  |
| rs1372045 | 11 | 76240662 |  | GWAS |  |
| rs4379869 | 11 | 76315299 |  | GWAS |  |
| rs1945465 | 11 | 78034146 |  | AIM |  |
| rs10501474 | 11 | 80078295 |  | AIM |  |
| rs2077815 | 11 | 85350031 | *PICALM* | Gene |  |
| rs510566 | 11 | 85355487 | *PICALM* | Gene |  |
| rs10501602 | 11 | 85359037 | *PICALM* | Gene |  |
| rs10501604 | 11 | 85361202 | *PICALM* | Gene |  |
| rs532470 | 11 | 85363744 | *PICALM* | Gene |  |
| rs713346 | 11 | 85365857 | *PICALM* | Gene |  |
| rs615887 | 11 | 85367689 | *PICALM* | Gene |  |
| rs669336 | 11 | 85376515 | *PICALM* | Gene |  |
| rs680119 | 11 | 85393680 | *PICALM* | Gene |  |
| rs642949 | 11 | 85407020 | *PICALM* | Gene |  |
| rs664629 | 11 | 85429531 | *PICALM* | Gene |  |
| rs1941375 | 11 | 85434781 | *PICALM* | Gene |  |
| rs597446 | 11 | 85452707 | *PICALM* | Gene |  |
| rs10898433 | 11 | 85458230 | *PICALM* | Gene |  |
| rs669556 | 11 | 85458970 | *PICALM* | Gene |  |
| rs621942 | 11 | 85461386 | *PICALM* | Gene |  |
| rs989088 | 11 | 93385286 | *HEPHL1* | Gene |  |
| rs1518560 | 11 | 93393270 | *HEPHL1* | Gene |  |
| rs1518561 | 11 | 93396497 | *HEPHL1* | Gene |  |
| rs11020631 | 11 | 93398541 | *HEPHL1* | Gene |  |
| rs1914723 | 11 | 93400154 | *HEPHL1* | Gene |  |
| rs2460055 | 11 | 93403799 | *HEPHL1* | Gene |  |
| rs1518566 | 11 | 93406232 | *HEPHL1* | Gene |  |
| rs4753116 | 11 | 93407008 | *HEPHL1* | Gene |  |
| rs12362164 | 11 | 93415789 | *HEPHL1* | Gene |  |
| rs2511386 | 11 | 93417356 | *HEPHL1* | Gene |  |
| rs7127348 | 11 | 93427406 | *HEPHL1* | Gene |  |
| rs10831161 | 11 | 93427918 | *HEPHL1* | Gene |  |
| rs2460042 | 11 | 93432783 | *HEPHL1* | Gene |  |
| rs4753122 | 11 | 93436811 | *HEPHL1* | Gene |  |
| rs4753534 | 11 | 93438521 | *HEPHL1* | Gene |  |
| rs2511410 | 11 | 93445262 | *HEPHL1* | Gene |  |
| rs2226927 | 11 | 93448510 | *HEPHL1* | Gene |  |
| rs2945637 | 11 | 93449266 | *HEPHL1* | Gene |  |
| rs2949858 | 11 | 93456570 | *HEPHL1* | Gene |  |
| rs2949856 | 11 | 93461272 | *HEPHL1* | Gene |  |
| rs4753124 | 11 | 93462883 | *HEPHL1* | Gene |  |
| rs10765653 | 11 | 93473094 | *HEPHL1* | Gene |  |
| rs7927016 | 11 | 93473871 | *HEPHL1* | Gene |  |
| rs4625415 | 11 | 93480352 | *HEPHL1* | Gene |  |
| rs4531426 | 11 | 93480506 | *HEPHL1* | Gene |  |
| rs10437575 | 11 | 93482465 | *HEPHL1* | Gene |  |
| rs7925817 | 11 | 93483682 | *HEPHL1* | Gene |  |
| rs7108501 | 11 | 93485000 | *HEPHL1* | Gene |  |
| rs567992 | 11 | 105767607 |  | AIM |  |
| rs990949 | 12 | 3356843 |  | AIM |  |
| rs4625554 | 12 | 4286565 |  | AIM |  |
| rs11054130 | 12 | 7521845 | *CD163* | Gene |  |
| rs10845265 | 12 | 7522100 | *CD163* | Gene |  |
| rs6488336 | 12 | 7523150 | *CD163* | Gene |  |
| rs6488338 | 12 | 7523410 | *CD163* | Gene |  |
| rs10743939 | 12 | 7529287 | *CD163* | Gene |  |
| rs4883263 | 12 | 7540751 | *CD163* | Gene |  |
| rs7954492 | 12 | 7545584 | *CD163* | Gene |  |
| rs11836971 | 12 | 7546545 | *CD163* | Gene |  |
| rs11054197 | 12 | 7548589 | *CD163* | Gene |  |
| rs1548837 | 12 | 12945584 |  | AIM |  |
| rs1861577 | 12 | 16455240 |  | AIM |  |
| rs14132 | 12 | 19565448 |  | AIM |  |
| rs706804 | 12 | 49661333 | *SLC11A2* | Gene |  |
| rs17125155 | 12 | 49661585 | *SLC11A2* | Gene |  |
| rs829021 | 12 | 49662086 | *SLC11A2* | Gene |  |
| rs853235 | 12 | 49662236 | *SLC11A2* | Gene |  |
| rs149411 | 12 | 49666499 | *SLC11A2* | Gene |  |
| rs2301529 | 12 | 49676014 | *SLC11A2* | Gene |  |
| rs12227734 | 12 | 49678590 | *SLC11A2* | Gene |  |
| rs224589 | 12 | 49685317 | *SLC11A2* | Gene |  |
| rs224572 | 12 | 49702787 | *SLC11A2* | Gene |  |
| rs337514 | 12 | 61538458 |  | AIM |  |
| rs4762106 | 12 | 64304740 |  | AIM |  |
| rs1433251 | 12 | 71362298 |  | AIM |  |
| rs1396226 | 12 | 73586112 |  | AIM |  |
| rs10506816 | 12 | 78448988 |  | AIM |  |
| rs1163016 | 12 | 79554821 |  | AIM |  |
| rs2051713 | 12 | 89479599 |  | AIM |  |
| rs249847 | 12 | 97391847 |  | AIM |  |
| rs1826734 | 12 | 101652738 |  | AIM |  |
| rs1861809 | 12 | 108708308 |  | AIM |  |
| rs903770 | 12 | 115754677 |  | AIM |  |
| rs4076700 | 12 | 115867703 |  | AIM |  |
| rs1179992 | 12 | 119958152 |  | AIM |  |
| rs918044 | 12 | 125402711 |  | AIM |  |
| rs4034627 | 12 | 126963425 |  | AIM |  |
| rs7136901 | 12 | 127679698 |  | GWAS |  |
| rs6486532 | 12 | 129261499 |  | AIM |  |
| rs1888057 | 13 | 32520695 |  | GWAS |  |
| rs1536289 | 13 | 58781783 |  | AIM |  |
| rs310935 | 13 | 61931782 |  | AIM |  |
| rs10507688 | 13 | 62304229 |  | AIM |  |
| rs188481 | 13 | 62710392 |  | AIM |  |
| rs7321723 | 13 | 76283962 |  | GWAS |  |
| rs767778 | 13 | 79095116 |  | AIM |  |
| rs5000507 | 13 | 80986955 |  | AIM |  |
| rs898271 | 13 | 90539922 |  | AIM |  |
| rs1408209 | 13 | 92805575 |  | AIM |  |
| rs10492585 | 13 | 104184177 |  | AIM |  |
| rs719185 | 13 | 107079922 |  | AIM |  |
| rs1010172 | 13 | 108644009 |  | AIM |  |
| rs9323178 | 14 | 22183486 |  | AIM |  |
| rs179562 | 14 | 30294209 |  | AIM |  |
| rs1451928 | 14 | 47410491 |  | AIM |  |
| rs1947393 | 14 | 49052346 |  | AIM |  |
| rs2224835 | 14 | 53476772 | *BMP4* | Gene |  |
| rs1951867 | 14 | 53476942 | *BMP4* | Gene |  |
| rs7146040 | 14 | 53477670 | *BMP4* | Gene |  |
| rs4444235 | 14 | 53480669 | *BMP4* | Gene |  |
| rs17563 | 14 | 53487272 | *BMP4* | Gene |  |
| rs762642 | 14 | 53492803 | *BMP4* | Gene |  |
| rs1951868 | 14 | 53495585 | *BMP4* | Gene |  |
| rs4901474 | 14 | 53539487 |  | Literature |  |
| rs311848 | 14 | 58270833 |  | AIM |  |
| rs2301106 | 14 | 61236316 | *HIF1A* | Gene |  |
| rs1951795 | 14 | 61241179 | *HIF1A* | Gene |  |
| rs7156573 | 14 | 61247574 | *HIF1A* | Gene |  |
| rs10144011 | 14 | 61261235 | *HIF1A* | Gene |  |
| rs1957757 | 14 | 61266701 | *HIF1A* | Gene |  |
| rs966824 | 14 | 61270271 | *HIF1A* | Gene |  |
| rs10873142 | 14 | 61273215 | *HIF1A* | Gene |  |
| rs2301113 | 14 | 61276301 | *HIF1A* | Gene |  |
| rs11549465 | 14 | 61277310 | *HIF1A* | Gene |  |
| rs2057482 | 14 | 61283601 | *HIF1A* | Gene |  |
| rs6573399 | 14 | 61285478 | *HIF1A* | Gene |  |
| rs1319462 | 14 | 61288978 | *HIF1A* | Gene |  |
| rs2302591 | 14 | 75178175 |  | GWAS |  |
| rs10131076 | 14 | 79844138 |  | AIM |  |
| rs981270 | 14 | 85249374 |  | AIM |  |
| rs4778137 | 15 | 26001430 |  | AIM |  |
| rs2873 | 15 | 29018547 |  | AIM |  |
| rs1524876 | 15 | 29050564 |  | AIM |  |
| rs12915800 | 15 | 32751988 |  | GWAS |  |
| rs1108081 | 15 | 35893455 |  | AIM |  |
| rs573615 | 15 | 41401573 |  | AIM |  |
| rs16966334 | 15 | 42790406 | *B2M* | Gene |  |
| rs2255235 | 15 | 42790656 | *B2M* | Gene |  |
| rs1690313 | 15 | 42792952 | *B2M* | Gene |  |
| rs2254835 | 15 | 42793910 | *B2M* | Gene |  |
| rs4780 | 15 | 42797314 | *B2M* | Gene |  |
| rs1648282 | 15 | 43213156 |  | AIM |  |
| rs1648312 | 15 | 43244641 |  | AIM |  |
| rs586799 | 15 | 45812214 |  | GWAS |  |
| rs1439323 | 15 | 45860350 |  | GWAS |  |
| rs9302185 | 15 | 52742156 |  | AIM |  |
| rs1037958 | 15 | 55310834 |  | AIM |  |
| rs387812 | 15 | 56862078 |  | AIM |  |
| rs936013 | 15 | 58505557 |  | AIM |  |
| rs11457 | 15 | 61673432 |  | AIM |  |
| rs13379875 | 15 | 71129834 | *NEO1* | Gene |  |
| rs4625692 | 15 | 71157520 | *NEO1* | Gene |  |
| rs4777589 | 15 | 71189956 | *NEO1* | Gene |  |
| rs16957640 | 15 | 71190747 | *NEO1* | Gene |  |
| rs4777590 | 15 | 71194141 | *NEO1* | Gene |  |
| rs8039533 | 15 | 71196954 | *NEO1* | Gene |  |
| rs7175329 | 15 | 71202934 | *NEO1* | Gene |  |
| rs7163504 | 15 | 71208606 | *NEO1* | Gene |  |
| rs1023924 | 15 | 71225675 | *NEO1* | Gene |  |
| rs11072404 | 15 | 71243024 | *NEO1* | Gene |  |
| rs7176908 | 15 | 71249454 | *NEO1* | Gene |  |
| rs1979409 | 15 | 71252530 | *NEO1* | Gene |  |
| rs4570800 | 15 | 71259858 | *NEO1* | Gene |  |
| rs1479340 | 15 | 71259940 | *NEO1* | Gene |  |
| rs2127277 | 15 | 71273104 | *NEO1* | Gene |  |
| rs17762600 | 15 | 71275806 | *NEO1* | Gene |  |
| rs747873 | 15 | 71315990 | *NEO1* | Gene |  |
| rs1871385 | 15 | 71316651 | *NEO1* | Gene |  |
| rs2251205 | 15 | 71338303 | *NEO1* | Gene |  |
| rs12903941 | 15 | 71341597 | *NEO1* | Gene |  |
| rs10518997 | 15 | 71343997 | *NEO1* | Gene |  |
| rs2660826 | 15 | 71344687 | *NEO1* | Gene |  |
| rs1131854 | 15 | 71351934 | *NEO1* | Gene |  |
| rs8027588 | 15 | 71352703 | *NEO1* | Gene |  |
| rs10438294 | 15 | 71363706 | *NEO1* | Gene |  |
| rs11072411 | 15 | 71365581 | *NEO1* | Gene |  |
| rs499098 | 15 | 71371837 | *NEO1* | Gene |  |
| rs2252725 | 15 | 71373320 | *NEO1* | Gene |  |
| rs2292915 | 15 | 71377632 | *NEO1* | Gene |  |
| rs531019 | 15 | 71385660 | *NEO1* | Gene |  |
| rs1394371 | 15 | 76511524 | *IREB2* | Gene |  |
| rs12903150 | 15 | 76511700 | *IREB2* | Gene |  |
| rs17483721 | 15 | 76520786 | *IREB2* | Gene |  |
| rs2568494 | 15 | 76528019 | *IREB2* | Gene |  |
| rs2656073 | 15 | 76529331 | *IREB2* | Gene |  |
| rs10519198 | 15 | 76529809 | *IREB2* | Gene |  |
| rs2656069 | 15 | 76532762 | *IREB2* | Gene |  |
| rs3817092 | 15 | 76551340 | *IREB2* | Gene |  |
| rs12904234 | 15 | 76566439 | *IREB2* | Gene |  |
| rs965604 | 15 | 76576278 | *IREB2* | Gene |  |
| rs1442307 | 15 | 85740218 |  | Literature |  |
| rs10520678 | 15 | 86738287 |  | AIM |  |
| rs4932370 | 15 | 89205709 | *FURIN* | Gene |  |
| rs4932178 | 15 | 89212660 | *FURIN* | Gene |  |
| rs17514846 | 15 | 89217554 | *FURIN* | Gene |  |
| rs4702 | 15 | 89227564 | *FURIN* | Gene |  |
| rs1894400 | 15 | 89229959 | *FURIN* | Gene |  |
| rs4932179 | 15 | 89231911 | *FURIN* | Gene |  |
| rs1266490 | 15 | 89258224 |  | AIM |  |
| rs1075840 | 15 | 89602911 |  | AIM |  |
| rs4965825 | 15 | 99668442 |  | GWAS |  |
| rs4785966 | 16 | 4458755 | *HMOX2* | Gene |  |
| rs4785969 | 16 | 4476935 | *HMOX2* | Gene |  |
| rs1362626 | 16 | 4489227 | *HMOX2* | Gene |  |
| rs1051308 | 16 | 4500302 | *HMOX2* | Gene |  |
| rs8129 | 16 | 4502352 | *HMOX2* | Gene |  |
| rs1004704 | 16 | 47094922 |  | AIM |  |
| rs2063099 | 16 | 50018372 |  | AIM |  |
| rs4784276 | 16 | 51542278 |  | GWAS |  |
| rs10500505 | 16 | 63500527 |  | AIM |  |
| rs461785 | 16 | 64366545 |  | AIM |  |
| rs1424241 | 16 | 70636408 | *HP* | Gene |  |
| rs6499557 | 16 | 70640155 | *HP* | Gene |  |
| rs5467 | 16 | 70645781 | *HP* | Gene |  |
| rs4130513 | 16 | 77016251 |  | AIM |  |
| rs2967305 | 16 | 80877154 |  | AIM |  |
| rs276990 | 16 | 84778717 |  | AIM |  |
| rs6503211 | 17 | 9333425 |  | AIM |  |
| rs987189 | 17 | 14491981 |  | GWAS |  |
| rs10491097 | 17 | 19301803 |  | AIM |  |
| rs7216657 | 17 | 23736516 | *SLC46A1* | Gene |  |
| rs9894260 | 17 | 23743752 | *SLC46A1* | Gene |  |
| rs1128162 | 17 | 23745918 | *SLC46A1* | Gene |  |
| rs2239910 | 17 | 23747793 | *SLC46A1* | Gene |  |
| rs739439 | 17 | 23747949 | *SLC46A1* | Gene |  |
| rs2239908 | 17 | 23749392 | *SLC46A1* | Gene |  |
| rs2239907 | 17 | 23749871 | *SLC46A1* | Gene |  |
| rs4795436 | 17 | 23753555 | *SLC46A1* | Gene |  |
| rs6505081 | 17 | 23762015 | *SLC46A1* | Gene |  |
| rs6503653 | 17 | 37117427 | *GAST* | Gene |  |
| rs7217461 | 17 | 37119282 | *GAST* | Gene |  |
| rs7219746 | 17 | 37121495 | *GAST* | Gene |  |
| rs9900213 | 17 | 37629407 | *STAT5B* | Gene |  |
| rs6503691 | 17 | 37647616 | *STAT5B* | Gene |  |
| rs16967611 | 17 | 37655093 | *STAT5B* | Gene |  |
| rs34129849 | 17 | 37672482 | *STAT5B* | Gene |  |
| rs7218653 | 17 | 37678846 | *STAT5B* | Gene |  |
| rs962272 | 17 | 44333282 |  | AIM |  |
| rs12150511 | 17 | 52006359 |  | GWAS |  |
| rs2253624 | 17 | 67243676 |  | AIM |  |
| rs11664524 | 18 | 7222892 |  | AIM |  |
| rs1013459 | 18 | 11690534 |  | AIM |  |
| rs2164062 | 18 | 17233261 |  | AIM |  |
| rs9962466 | 18 | 29418125 |  | GWAS |  |
| rs2042762 | 18 | 33531620 |  | AIM |  |
| rs11082698 | 18 | 44512434 |  | GWAS |  |
| rs7244227 | 18 | 46803943 | *SMAD4* | Gene |  |
| rs4390682 | 18 | 46806451 | *SMAD4* | Gene |  |
| rs12457540 | 18 | 46816802 | *SMAD4* | Gene |  |
| rs3764465 | 18 | 46825370 | *SMAD4* | Gene |  |
| rs8096092 | 18 | 46835599 | *SMAD4* | Gene |  |
| rs1792737 | 18 | 51997365 |  | AIM |  |
| rs9948708 | 18 | 58016101 |  | GWAS |  |
| rs12953952 | 18 | 65888907 |  | AIM |  |
| rs2660917 | 18 | 66779058 |  | Literature |  |
| rs1020382 | 19 | 218039 |  | AIM |  |
| rs2108389 | 19 | 3542590 |  | AIM |  |
| rs661821 | 19 | 7488649 | *MCOLN1* | Gene |  |
| rs612862 | 19 | 7499589 | *MCOLN1* | Gene |  |
| rs625910 | 19 | 7500159 | *MCOLN1* | Gene |  |
| rs604959 | 19 | 7505735 | *MCOLN1* | Gene |  |
| rs541271 | 19 | 7508039 | *MCOLN1* | Gene |  |
| rs2967890 | 19 | 12901647 | *CALR* | Gene |  |
| rs1010222 | 19 | 12909608 | *CALR* | Gene |  |
| rs12459782 | 19 | 18357495 | *GDF15* | Gene |  |
| rs1054564 | 19 | 18360815 | *GDF15* | Gene |  |
| rs16982345 | 19 | 18361722 | *GDF15* | Gene |  |
| rs6512265 | 19 | 18363835 | *GDF15* | Gene |  |
| rs888861 | 19 | 40073692 |  | AIM |  |
| rs1882694 | 19 | 40463222 | *HAMP* | Gene |  |
| rs7251432 | 19 | 40467281 | *HAMP* | Gene |  |
| rs12971321 | 19 | 40471262 | *HAMP* | Gene |  |
| rs268666 | 19 | 45610005 |  | AIM |  |
| rs1805419 | 19 | 54150916 | *FTL* | Gene |  |
| rs4645887 | 19 | 54151688 | *FTL* | Gene |  |
| rs1010104 | 19 | 54152727 | *FTL* | Gene |  |
| rs2387583 | 19 | 54153117 | *FTL* | Gene |  |
| rs905238 | 19 | 54157196 | *FTL* | Gene |  |
| rs1039442 | 19 | 54163142 | *FTL* | Gene |  |
| rs1042265 | 19 | 54163632 | *FTL* | Gene |  |
| rs12610125 | 19 | 54192994 |  | GWAS |  |
| rs6047134 | 20 | 2089054 |  | AIM |  |
| rs235711 | 20 | 6688089 | *BMP2* | Gene |  |
| rs235710 | 20 | 6688366 | *BMP2* | Gene |  |
| rs1980499 | 20 | 6694498 | *BMP2* | Gene |  |
| rs1979855 | 20 | 6695607 | *BMP2* | Gene |  |
| rs7270163 | 20 | 6699316 | *BMP2* | Gene |  |
| rs235764 | 20 | 6702246 | *BMP2* | Gene |  |
| rs235767 | 20 | 6703598 | *BMP2* | Gene |  |
| rs1005464 | 20 | 6704148 | *BMP2* | Gene |  |
| rs3178250 | 20 | 6708201 | *BMP2* | Gene |  |
| rs235770 | 20 | 6709765 | *BMP2* | Gene |  |
| rs28488 | 20 | 6710221 | *BMP2* | Gene |  |
| rs6117432 | 20 | 6712536 | *BMP2* | Gene |  |
| rs173107 | 20 | 6713841 | *BMP2* | Gene |  |
| rs235756 | 20 | 6715111 |  | Literature |  |
| rs708915 | 20 | 8348667 |  | AIM |  |
| rs747398 | 20 | 15497323 |  | AIM |  |
| rs11700002 | 20 | 24539936 |  | GWAS |  |
| rs1043415 | 20 | 35378652 |  | AIM |  |
| rs932428 | 20 | 37310186 |  | GWAS |  |
| rs1981431 | 20 | 43408865 |  | AIM |  |
| rs354747 | 20 | 58346055 |  | AIM |  |
| rs354731 | 20 | 58384823 |  | AIM |  |
| rs6090153 | 20 | 60991628 |  | GWAS |  |
| rs816943 | 20 | 62146453 |  | AIM |  |
| rs2825717 | 21 | 19950789 |  | GWAS |  |
| rs2829454 | 21 | 25194942 |  | AIM |  |
| rs2300301 | 21 | 29598187 | *BACH1* | Gene |  |
| rs1153280 | 21 | 29599943 | *BACH1* | Gene |  |
| rs1153284 | 21 | 29604904 | *BACH1* | Gene |  |
| rs1153285 | 21 | 29605821 | *BACH1* | Gene |  |
| rs1236481 | 21 | 29619544 | *BACH1* | Gene |  |
| rs411697 | 21 | 29636469 | *BACH1* | Gene |  |
| rs425989 | 21 | 29637146 | *BACH1* | Gene |  |
| rs368322 | 21 | 29639022 | *BACH1* | Gene |  |
| rs382732 | 21 | 29640881 | *BACH1* | Gene |  |
| rs117214 | 21 | 29642705 | *BACH1* | Gene |  |
| rs2832286 | 21 | 29642842 | *BACH1* | Gene |  |
| rs2832291 | 21 | 29656259 | *BACH1* | Gene |  |
| rs2837888 | 21 | 41257856 |  | AIM |  |
| rs2837956 | 21 | 41401386 |  | AIM |  |
| rs4821790 | 22 | 20841370 |  | GWAS |  |
| rs987710 | 22 | 20842415 |  | GWAS |  |
| rs361959 | 22 | 21199742 |  | GWAS tag SNP |  |
| rs738402 | 22 | 25007934 |  | AIM |  |
| rs5995096 | 22 | 34099966 | *HMOX1* | Gene |  |
| rs2071748 | 22 | 34107618 | *HMOX1* | Gene |  |
| rs2071749 | 22 | 34113413 | *HMOX1* | Gene |  |
| rs11912889 | 22 | 34113617 | *HMOX1* | Gene |  |
| rs5755720 | 22 | 34116873 | *HMOX1* | Gene |  |
| rs2285112 | 22 | 34119263 | *HMOX1* | Gene |  |
| rs743811 | 22 | 34122974 | *HMOX1* | Gene |  |
| rs1534880 | 22 | 35653611 |  | AIM |  |
| rs131843 | 22 | 35667355 |  | Literature |  |
| rs760719 | 22 | 35790395 | *TMPRSS6* | Gene |  |
| rs855791 | 22 | 35792882 | *TMPRSS6* | Gene and Literature |  |
| rs5756504 | 22 | 35797216 | *TMPRSS6* | Gene |  |
| rs5756505 | 22 | 35797300 | *TMPRSS6* | Gene |  |
| rs4820268 | 22 | 35799537 | *TMPRSS6* | Gene and Literature |  |
| rs2413450 | 22 | 35800170 | *TMPRSS6* | Gene |  |
| rs855788 | 22 | 35804025 | *TMPRSS6* | Gene |  |
| rs1005478 | 22 | 35808200 | *TMPRSS6* | Gene |  |
| rs7286184 | 22 | 35808721 | *TMPRSS6* | Gene |  |
| rs2111833 | 22 | 35810743 | *TMPRSS6* | Gene |  |
| rs2235324 | 22 | 35815670 | *TMPRSS6* | Gene |  |
| rs1421312 | 22 | 35817756 | *TMPRSS6* | Gene |  |
| rs2743825 | 22 | 35822147 | *TMPRSS6* | Gene |  |
| rs2160906 | 22 | 35823124 | *TMPRSS6* | Gene |  |
| rs732756 | 22 | 35824366 | *TMPRSS6* | Gene |  |
| rs733655 | 22 | 35824997 | *TMPRSS6* | Gene |  |
| rs228906 | 22 | 35825768 | *TMPRSS6* | Gene |  |
| rs228907 | 22 | 35827539 | *TMPRSS6* | Gene |  |
| rs17750152 | 22 | 35827972 | *TMPRSS6* | Gene |  |
| rs4140589 | 22 | 35828965 | *TMPRSS6* | Gene |  |
| rs228909 | 22 | 35830589 | *TMPRSS6* | Gene |  |
| rs228910 | 22 | 35831016 | *TMPRSS6* | Gene |  |
| rs228913 | 22 | 35834103 | *TMPRSS6* | Gene |  |
| rs138022 | 22 | 38942982 |  | AIM |  |
| rs1007321 | X | 21727592 |  | AIM |  |
| rs1884688 | X | 37260332 |  | AIM |  |
| rs743151 | X | 37784743 |  | AIM |  |
| rs4076107 | X | 39696224 |  | AIM |  |
| rs953114 | X | 40953796 |  | AIM |  |
| rs704145 | X | 55052008 | *ALAS2* | Gene |  |
| rs1476012 | X | 55061869 | *ALAS2* | Gene |  |
| rs1011526 | X | 65199108 |  | AIM |  |
| rs1264216 | X | 65218278 |  | AIM |  |
| rs4892539 | X | 74215399 | *ABCB7* | Gene |  |
| rs5937939 | X | 74215732 | *ABCB7* | Gene |  |
| rs6647627 | X | 74233608 | *ABCB7* | Gene |  |
| rs6647628 | X | 74236889 | *ABCB7* | Gene |  |
| rs1954279 | X | 74237503 | *ABCB7* | Gene |  |
| rs5937944 | X | 74247021 | *ABCB7* | Gene |  |
| rs4148837 | X | 74251412 | *ABCB7* | Gene |  |
| rs6647631 | X | 74252731 | *ABCB7* | Gene |  |
| rs2153814 | X | 74262663 | *ABCB7* | Gene |  |
| rs5981775 | X | 74280620 | *ABCB7* | Gene |  |
| rs1935074 | X | 79983248 |  | AIM |  |
| rs525869 | X | 90402304 |  | AIM |  |
| rs2335011 | X | 97632889 |  | GWAS |  |
| rs1152324 | X | 106099634 |  | AIM |  |
| rs980099 | X | 106118132 |  | AIM |  |
| rs537111 | X | 108534073 |  | AIM |  |
| rs2016878 | X | 109740071 |  | AIM |  |
| rs1558022 | X | 116290201 |  | AIM |  |
| rs2040962 | X | 116385288 |  | AIM |  |
| rs2430212 | X | 116972084 |  | Literature |  |
| rs1716758 | X | 117241790 |  | AIM |  |
| rs2428754 | X | 118244599 | *PGRMC1* | Gene |  |
| rs12558236 | X | 118247834 | *PGRMC1* | Gene |  |
| rs1934070 | X | 121103292 |  | AIM |  |
| rs7881297 | X | 121338454 |  | AIM |  |
| rs1883906 | X | 126414779 |  | AIM |  |
| rs2200290 | X | 126713002 |  | AIM |  |
| rs1005488 | X | 131116515 |  | AIM |  |
| rs5933503 | X | 134041583 |  | GWAS |  |
| rs1487921 | X | 137133858 |  | AIM |  |
| rs2485729 | X | 138707739 |  | AIM |  |
| rs6540401 | X | 147024359 |  | AIM |  |
| rs758439 | X | 147770441 |  | AIM |  |
| rs530501 | X | 148273210 |  | AIM |  |
| rs5925535 | X | 149290241 |  | GWAS |  |
| rs1882719 | X | 150272071 |  | AIM |  |
