# Supplementary material for: Associations between Single Nucleotide Polymorphisms in Iron-Related Genes and Iron Status in Multiethnic Populations
Source: PLoS One. 2012 Jun 22;7(6):e38339. doi: 10.1371/journal.pone.0038339 (PMC3382217; doi:10.1371/journal.pone.0038339)
Supplement: Table S2 — Descriptive statistics by population, iron deficient case-control status, and sex. (DOC) [file pone.0038339.s002.doc]

**Table S2. Descriptive statistics by population, iron deficient case-control status, and sex.**

|  |  | | | |  | | | |  | | | |  | | | |
| --- | --- | --- | --- | --- | --- | --- | --- | --- | --- | --- | --- | --- | --- | --- | --- | --- |
| **Variable^a^** | **White (N=1067)** | | | | **African American (N=212)** | | | | **Hispanic (N=233)** | | | | **Asian (N=147)** | | | |
|  | **Case** | | **Control** | | **Case** | | **Control** | | **Case** | | **Control** | | **Case** | | **Control** | |
|  |  | |  | |  | |  | |  | |  | |  | |  | |
| Total Sample Size | 352 | | 715 | | 74 | | 138 | | 77 | | 156 | | 51 | | 96 | |
|  |  | |  | |  | |  | |  | |  | |  | |  | |
|  | Male | Female | Male | Female | Male | Female | Male | Female | Male | Female | Male | Female | Male | Female | Male | Female |
| Sample size | 83 | 269 | 171 | 544 | 25 | 49 | 52 | 86 | 19 | 58 | 40 | 116 | 18 | 33 | 35 | 61 |
|  |  |  |  |  |  |  |  |  |  |  |  |  |  |  |  |  |
| Body Iron | -2.4 (2.34) | -1.9 (2.48) | 12.5 (2.19) | 10.2 (2.42) | -4.9 (2.80) | -4.0 (3.40) | 11.8 (2.39) | 10.2 (2.73) | -3.8 (2.85) | -3.5 (3.28) | 12.9 (2.46) | 10.2 (2.49) | -3.1 (2.81) | -3.5 (3.03) | 13.4 (2.70) | 11.7 (2.26) |
|  |  |  |  |  |  |  |  |  |  |  |  |  |  |  |  |  |
| SF (μg/L) | 8.1 (2.02) | 7.9 (2.38) | 271.7 (167.41) | 143.9 (123.27) | 6.5 (2.35) | 6.5 (2.56) | 296.7 (266.06) | 186.7 (169.96) | 6.2 (1.70) | 5.6 (2.33) | 325.5 (571.37) | 140.6 (115.51) | 6.9 (2.50) | 5.6 (1.80) | 327.4 (177.94) | 198.0 (107.16) |
|  |  |  |  |  |  |  |  |  |  |  |  |  |  |  |  |  |
| TfS (%) | 12.8 (8.10) | 13.4 (7.37) | 33.2 (12.03) | 28.3 (10.20) | 8.5 (5.22) | 10.7 (6.96) | 32.7 (14.99) | 24.5 (9.00) | 10.9 (7.71) | 11.8 (6.63) | 34.7 (13.95) | 25.7 (9.10) | 10.7 (6.54) | 10.8 (7.32) | 38.4 (15.78) | 32.7 (10.34) |
|  |  |  |  |  |  |  |  |  |  |  |  |  |  |  |  |  |
| sTfR (μg/L) | 7.5 (3.71) | 5.7 (3.04) | 3.1 (0.90) | 3.0 (0.98) | 11.6 (5.93) | 9.7 (7.36) | 4.0 (1.38) | 3.9 (1.54) | 8.7 (5.39) | 6.4 (4.06) | 2.7 (0.83) | 2.9 (1.02) | 7.5 (4.13) | 7.0 (4.67) | 3.1 (1.28) | 3.1 (2.71) |
|  |  |  |  |  |  |  |  |  |  |  |  |  |  |  |  |  |
| Serum Iron (μg/dL) | 48.7 (27.60) | 53.2 (27.76) | 98.5 (32.26) | 88.2 (29.16) | 33.6 (19.28) | 42.7 (27.09) | 89.1 (30.50) | 73.3 (26.87) | 44.9 (33.89) | 48.4 (25.62) | 102.8 (31.43) | 80.9 (28.89) | 42.5 (25.66) | 43.8 (28.34) | 109.1 (39.84) | 99.5 (24.45) |
|  |  |  |  |  |  |  |  |  |  |  |  |  |  |  |  |  |
| TIBC (μg/dL) | 393.5 (48.93) | 403.5 (53.33) | 302.0 (41.14) | 317.4 (45.60) | 410.4 (40.44) | 405.4 (49.38) | 285.5 (43.83) | 303.4 (43.07) | 416.8 (52.49) | 421.0 (56.89) | 303.8 (37.22) | 316.5 (40.87) | 398.2 (40.47) | 414.9 (46.53) | 289.4 (36.50) | 311.0 (37.20) |
|  |  |  |  |  |  |  |  |  |  |  |  |  |  |  |  |  |
| UIBC (μg/dL) | 344.8 (62.82) | 350.3 (62.87) | 203.4 (51.06) | 229.2 (52.37) | 376.8 (48.50) | 362.7 (57.74) | 196.4 (57.44) | 226.6 (57.93) | 371.9 (60.23) | 372.5 (64.59) | 201.0 (54.04) | 235.6 (46.44) | 355.7 (46.27) | 371.1 (59.09) | 180.2 (54.73) | 211.5 (48.23) |

^a^Values shown are the mean (standard deviation) for quantitative variables.
