# Supplementary material for: Associations between Single Nucleotide Polymorphisms in Iron-Related Genes and Iron Status in Multiethnic Populations
Source: PLoS One. 2012 Jun 22;7(6):e38339. doi: 10.1371/journal.pone.0038339 (PMC3382217; doi:10.1371/journal.pone.0038339)
Supplement: Table S3 — Forty-nine SNPs that showed statistically significant p-values, corrected for multiple testing, for at least one of eight iron-related outcomes in the white sample (48 SNPS) or the African-American sample (one SNP). (DOCX) [file pone.0038339.s003.docx]

Table S3. Forty-nine SNPs that showed statistically significant p-values, corrected for multiple testing, for at least one of eight iron-related outcomes in the white sample (48 SNPS) or the African-American sample (one SNP)^a^

|  |  |  |  | SNP  selection^c^ | Multiple Regression Model Outcome^b^ | | | | | | | |
| --- | --- | --- | --- | --- | --- | --- | --- | --- | --- | --- | --- | --- |
| SNP  Name | Chr | Position | Gene |  | Case | BI | Log_e_(SF) | TfS | Log_e_(TfR) | SI | UIBC | TIBC |
| rs164176 | 1 | 160622113 |  | GWAS | 0.0035 | 0.0057 | 0.0020 | 0.0210 | 0.1053 | 0.1141 | 0.0001 | **4.09x10^-5^** |
| rs1816618 | 2 | 64301388 |  | GWAS | 0.0023 | 0.0011 | 0.0030 | 4.84x10^-5^ | 0.0014 | 0.0037 | **3.46x10^-5^** | 0.0002 |
| rs871776 | 2 | 64309178 |  | GWAS | 0.0037 | 0.0018 | 0.0040 | 0.0002 | 0.0031 | 0.0130 | **3.64x10^-5^** | 0.0001 |
| rs2698524 | 2 | 64324864 |  | tag SNP | 0.0005 | 0.0003 | 0.0007 | **1.03x10^-5^** | 0.0016 | 0.0006 | **9.67x10^-6^** | 0.0001 |
| rs6750096 | 2 | 64327573 |  | GWAS | 0.0035 | 0.0018 | 0.0042 | 0.0002 | 0.0029 | 0.0133 | **3.69x10^-5^** | 0.0001 |
| rs2251764 | 2 | 64338969 |  | tag SNP | 0.0004 | 0.0002 | 0.0005 | **3.24x10^-5^** | 0.0005 | 0.0060 | **4.04x10^-6^** | **8.48x10^-6^** |
| rs2698541 | 2 | 64342183 |  | GWAS | 0.0002 | 0.0001 | 0.0002 | **1.83x10^-5^** | 0.0002 | 0.0044 | **1.28x10^-6^** | **2.62x10^-6^** |
| rs2555442 | 2 | 64342795 |  | tag SNP | 0.0004 | 0.0002 | 0.0004 | **3.06x10^-5^** | 0.0004 | 0.0068 | **2.25x10^-6^** | **3.68x10^-6^** |
| rs890482 | 2 | 64346439 |  | tag SNP | 0.0003 | 0.0002 | 0.0004 | **2.68x10^-5^** | 0.0003 | 0.0061 | **2.97x10^-6^** | **5.72x10^-6^** |
| rs2698527 | 2 | 64353133 |  | tag SNP | 0.0004 | 0.0002 | 0.0006 | 0.0001 | 0.0003 | 0.0136 | **4.17x10^-6^** | **4.19x10^-6^** |
| rs1426710 | 2 | 64355179 |  | tag SNP | 0.0003 | 0.0001 | 0.0003 | **1.12x10^-5^** | 0.0006 | 0.0033 | **4.80x10^-6^** | **1.69x10^-5^** |
| rs2698530 | 2 | 64357399 |  | GWAS | 0.0002 | 0.0001 | 0.0003 | **6.01x10^-6^** | 0.0006 | 0.0013 | **7.05x10^-6^** | 0.0001 |
| rs1867504 | 3 | 134893351 |  | Literature | 0.2982 | 0.3942 | 0.4187 | 0.0781 | 0.4483 | 0.6111 | 0.0002 | **8.22x10^-6^** |
| rs4525863 | 3 | 134918826 |  | GWAS/Lit. | 0.7009 | 0.5943 | 0.3518 | 0.1741 | 0.8546 | 0.6248 | 0.0001 | **2.16x10^-7^** |
| rs4428180 | 3 | 134949064 | *TF* | Gene | 0.0569 | 0.2099 | 0.1547 | 0.0853 | 0.7649 | 0.9794 | **1.40x10^-5^** | **2.95x10^-8^** |
| rs8177224 | 3 | 134956693 | *TF* | Gene | 0.7317 | 0.8159 | 0.5507 | 0.1776 | 0.7043 | 0.4045 | **2.05x10^-5^** | **2.40x10^-9^** |
| rs3811658 | 3 | 134959542 | *TF* | Gene | 0.8019 | 0.9800 | 0.8424 | 0.1501 | 0.6623 | 0.2452 | **1.69x10^-7^** | **1.14x10^-13^** |
| rs8177248 | 3 | 134962316 | *TF* | Gene | 0.7001 | 0.9403 | 0.8087 | 0.0956 | 0.8060 | 0.2359 | **5.83x10^-8^** | **1.24x10^-14^** |
| rs1880669 | 3 | 134966386 | *TF* | Gene | 0.0513 | 0.1384 | 0.1284 | 0.0346 | 0.3234 | 0.8760 | **6.77x10^-8^** | **2.31x10^-12^** |
| rs3811647 | 3 | 134966719 | *TF* | GWAS/Gene  /Lit. | 0.6876 | 0.9419 | 0.7752 | 0.1387 | 0.7239 | 0.1733 | **6.99x10^-8^** | **5.02×10^-15^** |
| rs1358024 | 3 | 134966878 | *TF* | GWAS/Lit. | 0.0917 | 0.2314 | 0.5004 | 0.0329 | 0.0217 | 0.9959 | **8.13x10^-6^** | **1.00x10^-8^** |
| rs1525892 | 3 | 134967402 | *TF* | Gene | 0.6679 | 0.8689 | 0.7575 | 0.0715 | 0.9076 | 0.2624 | **3.03x10^-8^** | **4.56×10^-15^** |
| rs9824452 | 3 | 134975161 | *TF* | tag SNP | 0.4688 | 0.4250 | 0.6050 | 0.0130 | 0.1803 | 0.2512 | 0.0001 | **1.39x10^-5^** |
| rs7638018 | 3 | 134978151 | *TF* | Gene | 0.6441 | 0.8685 | 0.7351 | 0.0892 | 0.8827 | 0.2422 | **5.41x10^-8^** | **1.22×10^-14^** |
| rs4854760 | 3 | 134981431 |  | Gene | 0.6923 | 0.9367 | 0.7949 | 0.1054 | 0.8046 | 0.2024 | **7.12x10^-8^** | **1.29×10^-14^** |
| rs4854762 | 3 | 134981753 |  | tag SNP | 0.7742 | 0.9814 | 0.8141 | 0.1371 | 0.7450 | 0.1797 | **2.03x10^-7^** | **5.71×10^-14^** |
| rs9843728 | 3 | 134984097 | *SRPRB* | Gene | 0.8603 | 0.8988 | 0.8188 | 0.0905 | 0.9555 | 0.3522 | **2.45x10^-6^** | **6.23×10^-14^** |
| rs1830084 | 3 | 134991154 | *SRPRB* | tag SNP | 0.8670 | 0.8801 | 0.9943 | 0.1429 | 0.7942 | 0.2532 | **2.28x10^-6^** | **1.41x10^-11^** |
| rs6794676 | 3 | 135000689 | *SRPRB* | tag SNP | 0.6014 | 0.7397 | 0.7007 | 0.5876 | 0.0757 | 0.3572 | 0.0033 | **1.61x10^-5^** |
| rs6794945 | 3 | 135001153 | *SRPRB* | GWAS/Lit. | 0.8724 | 0.9241 | 0.8882 | 0.1669 | 0.6355 | 0.1553 | **1.01x10^-6^** | **8.83x10^-13^** |
| rs13061203 | 3 | 135001825 | *SRPRB* | tag SNP | 0.4793 | 0.5919 | 0.8284 | 0.6598 | 0.0379 | 0.2872 | 0.0065 | **3.78x10^-5^** |
| rs9853615 | 3 | 135002671 | *SRPRB* | tag SNP | 0.4901 | 0.4666 | 0.5810 | 0.0993 | 0.2579 | 0.9997 | 0.0004 | **8.30x10^-6^** |
| rs7745902 | 6 | 132878166 |  | GWAS | 0.0095 | 0.0019 | 0.0028 | 0.0171 | 0.0050 | 0.4613 | **3.43x10^-5^** | **5.42x10^-7^** |
| rs10263415^d^ | 7 | 7691330 | *RPA3*^d^ | GWAS | 0.0047 | 0.0044 | 0.0522 | 0.0003 | **3.42x10^-5^** | 0.0003 | 0.0038 | 0.0759 |
| rs4725084 | 7 | 8343391 |  | GWAS | 0.0013 | 0.0010 | 0.0032 | **9.67x10^-6^** | 0.0013 | 0.0016 | **1.68x10^-5^** | 0.0002 |
| rs2460970^d^ | 8 | 119698635 | *SAMD12* ^d^ | GWAS | **1.42x10^-6^** | **2.65x10^-5^** | **1.13x10^-5^** | 0.0003 | 0.0169 | 0.0011 | 0.0009 | 0.0132 |
| rs880034^d^ | 8 | 119702442 | *SAMD12* ^d^ | GWAS | **1.42x10^-6^** | **2.65x10^-5^** | **1.13x10^-5^** | 0.0003 | 0.0169 | 0.0011 | 0.0009 | 0.0132 |
| rs10904850 | 10 | 17037713 | *CUBN* | Gene | 0.0274 | 0.0312 | 0.0202 | 0.0011 | 0.1887 | **1.04x10^-5^** | 0.0090 | 0.1387 |
| rs1149580 | 11 | 76227161 |  | GWAS | 0.0007 | 0.0001 | 0.0010 | 0.0008 | 0.0004 | 0.1600 | **1.81x10^-5^** | **2.08x10^-6^** |
| rs1372045 | 11 | 76240662 |  | GWAS | 0.0001 | 0.0003 | 0.0017 | 0.0031 | 0.0005 | 0.3067 | 0.0002 | **2.23x10^-5^** |
| rs12915800 | 15 | 32751988 |  | GWAS | 0.0003 | 0.0001 | 0.0002 | 0.0002 | **1.32x10^-5^** | 0.0002 | 0.0004 | 0.0103 |
| rs9948708^d^ | 18 | 58016101 | *KIAA1468* ^d^ | GWAS | 0.0137 | 0.0053 | 0.0088 | 0.0032 | 0.0043 | 0.0390 | 4.52x10^-5^ | **2.90x10^-5^** |
| rs11700002^d^ | 20 | 24539936 | *TMEM90B* ^d^ | GWAS | 0.0117 | 0.0023 | 0.0029 | 0.1662 | 0.0020 | 0.6185 | 0.0008 | **4.41x10^-6^** |
| rs987710 | 22 | 20842415 |  | GWAS | **1.18x10^-6^** | **6.34x10^-7^** | **1.29x10^-6^** | 0.0022 | 0.0001 | 0.0085 | 0.0036 | 0.0155 |
| rs2111833 | 22 | 35810743 | *TMPRSS6* | Gene | 0.3991 | 0.2020 | 0.4653 | 0.0001 | 0.0294 | **4.66x10^-7^** | 0.0271 | 0.8991 |
| rs2235324 | 22 | 35815670 | *TMPRSS6* | Gene | 0.4048 | 0.1278 | 0.2924 | 0.0012 | 0.0233 | **2.36x10^-6^** | 0.0861 | 0.7430 |
| rs1421312 | 22 | 35817756 | *TMPRSS6* | Gene | 0.4708 | 0.1486 | 0.3088 | 0.0018 | 0.0284 | **3.71x10^-6^** | 0.1135 | 0.6546 |
| rs2743825 | 22 | 35822147 | *TMPRSS6* | Gene | 0.9070 | 0.3253 | 0.5522 | 0.0046 | 0.0645 | **3.44x10^-6^** | 0.3232 | 0.2146 |
| rs2335011 | X | 97632889 |  | GWAS | 0.0002 | 0.0001 | **2.27x10^-5^** | 0.0558 | 0.0099 | 0.8168 | 0.0024 | 0.0001 |

^a^ Forty-eight of 49 SNPS were significantly associated with at least one outcome in the white population sample; rs10904850 in the *CUBN* gene was significantly associated with serum iron in the African-American population sample.

^b^ Regression outcomes: case-control status (case), body iron, (BI) natural log of serum ferritin concentration (log_e_SF), transferrin saturation (TfS), natural log of serum transferrin receptor (log_e_sTfR), serum iron (SI), unsaturated iron-binding capacity (UIBC), and total iron-binding capacity (TIBC). Values in bold are less than the Bonferroni multiple test-corrected nominal p-value of 0.05/1134 = 4.4×10^-5^.

^c^ Reasons for selecting SNPs for analysis are given as “GWAS” for SNPs significantly associated with any of the eight outcomes in our previously preformed GWAS, “tag SNP” for SNPs tagging regions around SNPs that were significantly associated with one of the outcomes in our previous GWAS and replicated in an independent population, “Literature or Lit.” for SNPs reported in the literature as being associated with iron-related traits, and “Gene” for SNPs tagging candidate genes.

^d^ SNP identified through our GWAS but not known to be in iron-related genes.
